# Data-Driven Discovery and Experimental Validation of Solvent Polarity Effects on Conjugated Polymer Solution-to-Film Assembly Pathways

**DOI:** 10.1021/acs.chemmater.6c00358

**Published:** 2026-07-16

**Authors:** Myeongyeon Lee, Sanghyun Jeon, Yen-Chi Chen, Priyotosh Bairagya, Chiwon Yu, Azzaya Khasbaatar, Nahyun Ahn, Ying Diao, Martha A. Grover, Elsa Reichmanis

**Affiliations:** † Department of Chemical and Biomolecular Engineering, 1687 Lehigh University, Bethlehem, Pennsylvania 18015, United States; ‡ Department of Materials Science and Engineering, 14589 University of Illinois at Urbana-Champaign, 1304 West Green Street, Urbana, Illinois 61801, United States; § Department of Chemical and Biomolecular Engineering, University of Illinois at Urbana-Champaign, 600 South Mathews Avenue, Urbana, Illinois 61801, United States; ∥ School of Chemical and Biomolecular Engineering, 1372 Georgia Institute of Technology, 311 Ferst Drive, Atlanta, Georgia 30332, United States

## Abstract

Understanding how solvent properties influence the solution-to-film assembly of conjugated polymers remains a critical challenge due to the complex and intertwined nature of polymer–solvent interactions. In this study, we integrate a data-driven framework with experimental validation to identify key parameters influencing the assembly and performance of poly­[2,5-(2-octyldodecyl)-3,6-diketopyrrolopyrrole-*alt*-5,5-(2,5-di­(thien-2-yl)­thieno­[3,2-*b*]­thiophene)] (DPP-DTT) in organic field-effect transistors (OFETs). A machine learning (ML) approach identified the normalized Reichardt polarity parameter (E_T_^N^) as a significant descriptor correlated with DPP-DTT hole mobility (μ). Systematic DPP-DTT devices fabricated using solvents across a wide E_T_^N^ range revealed that higher E_T_^N^ solvents yield enhanced μ. To elucidate the structural origins of high μ, we conducted comprehensive analyses using UV–vis–NIR spectroscopy and grazing incidence wide angle X-ray scattering (GIWAXS) measurements. The results revealed that films processed from high E_T_^N^ solvents exhibit reduced paracrystallinity. By analyzing the solution-state behavior using optical microscopy and solution WAXS, we revealed polymer solubility differences in the various solvents and associated distinct polymer assembly pathways, elucidating why the high E_T_^N^ solvent produces long-range ordered films. Notably, the high E_T_^N^ solvent shows a pronounced preference for liquid-crystal (LC)-mediated assembly, providing a mechanistic explanation for the enhanced structural order. Therefore, these results demonstrate that solvent polarity, as evaluated by E_T_^N^, serves as an important parameter that plays a significant role in the DPP-DTT assembly pathway and resultant solid-state morphology. This work provides a strategy for integrating data science with experiments to identify critical parameters associated with complex polymer systems and helps guide rational process design for high-performance organic electronics.

## Graphical abstract

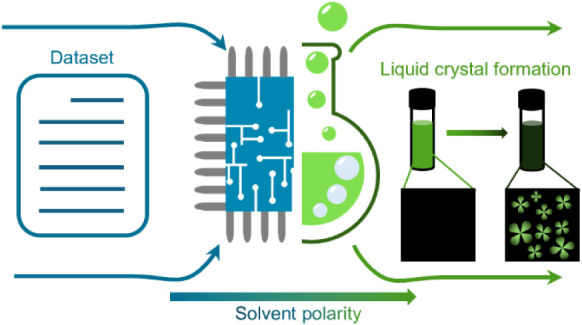

## Introduction

Conjugated polymers have received significant attention due to their versatile properties, which enable the fabrication of large-area,^1^ printable,^2^ and deformable electronic^3^ and energy storage and conversion devices such as OFETs,^4^ organic photovoltaics (OPVs),^5^ organic light-emitting diodes (OLEDs),^6^ and sensors.^7^ The performance of conjugated polymer-based devices is closely associated with their solid-state morphology, which arises from polymer chain packing in crystalline regions and the distribution of grain boundaries.^8^ In earlier studies, various strategies were explored to tune the solid-state morphology. In terms of intrinsic polymer properties, structural modifications of the polymers^9,10^ or adjustments in molecular weight have been investigated.^11,12^ From the solution processing perspective, solvent blending,^13^ deposition technique,^14^ and postdeposition processing^15^ have been reported to effectively control final thin-film morphology.

Although these studies have provided valuable insights into how individual factors influence the conjugated polymer assembly process and ultimate device performance, the overall relationship among polymer structure, processing conditions, solid-state morphology, and resultant device characteristics remains highly complex.^16^ The large number of intertwined parameters creates a vast and multidimensional design space that is challenging to explore. Furthermore, recent recognition of the important roles of polymer aggregation in solution^17–20^ and solution-to-film assembly pathways^21,22^ has added another layer of complexity to this research landscape. To address this complexity, data-driven approaches are beginning to emerge as effective means to identify key factors that influence polymer morphology and resultant device performance, thereby facilitating their experimental realization and optimization.^23–25^ For example, Venkatesh et al. applied a customized algorithm in conjunction with experiments to reveal that solution concentration is a key factor influencing OFET performance, with the critical overlap concentration corresponding to the optimal point for achieving high charge carrier mobility.^26^

While solvent properties and solvent–polymer interactions are known to play an important role in the solution-to-film assembly of conjugated polymers,^27,28^ the specific solvent properties affecting this assembly behavior remain elusive. This may be attributed to the fact that solvents exhibit multiple, often interrelated types of interactions with the solute, and that many solvent descriptors are strongly correlated, posing challenges to quantifying their overall effects with a single parameter.^29^ For instance, Hansen solubility parameters (HSPs) have been widely employed to describe polymer–solvent interactions; however, their applicability to conjugated polymers is often limited. Standard HSP theory considers only dispersive, polar, and hydrogen-bonding contributions based on group contribution theory, which are insufficient to capture the full spectrum of interactions in conjugated polymer systems. In particular, in these systems π–π interactions involve delocalized electronic states that play a dominant role in solution-state behavior but cannot be fully accounted for by group contribution theory.

Nevertheless, adopting a representative or alternative descriptor that reasonably captures these combined effects is crucial to effectively apply data-driven approaches and provide meaningful experimental guidelines. Among the various solvent parameters, E_T_^N^ is an empirical polarity scale derived from the solvatochromic shift of betaine dye which changes color dramatically depending on solvent polarity.^30^ Betaine dye is a relatively large, conjugated chromophore, raising the possibility that E_T_^N^ may also reflect solvent interactions relevant to conjugated polymer backbones. This metric has been widely reported for a broad range of solvents. E_T_^N^ reflects multiple solvent parameters, including dipolarity, polarizability, and hydrogen bond donating and accepting abilities, providing a comprehensive descriptor of solvent properties.^31–33^

In this study, we employed a data-driven framework integrating data science and experimental validation to identify key parameters that influence conjugated polymer assembly in the solution to solidified thin film states, and derive new mechanistic insights into factors to be considered in conjugated polymer design. We constructed a data set for DPP-DTT by extracting parameters such as polymer dispersity index (PDI), weight-average molecular weight (*M*_w_), solution concentration, insulating polymer blending (binary: performed/not performed), self-assembled monolayer (SAM) treatment (binary: applied/not applied), meniscus-guided coating (MGC) (binary: used/not used), annealing (binary: performed/not performed), poor solvent addition (binary: added/not added), and post chemical process (binary: performed/not performed), together with the corresponding experimental μ values from our previously established database^24^ and solvent E_T_^N^ values. Most importantly, the results identified E_T_^N^ as a parameter strongly correlated with OFET μ. Further, we revealed a mechanistic link between E_T_^N^, DPP-DTT aggregation state, the solution-to-film assembly process, and resultant device performance.

## Materials and Methods

### Data Science Methodology

#### Data Preparation

A total of 204 data points consisting of process parameters and corresponding charge mobilities were extracted from the database,^24^ including 135 data points from literature^7,26,34–49^ and 69 data points from experiments conducted by a former group member and a collaborating laboratory. To avoid multicollinearity and redundancy, those process parameters that had zero variance (all entries were 0), showed highly pairwise Pearson correlations with other parameters, were directly tied to the charge mobility equation, or described OFET device-structure parameters such as structural configuration, which influence μ but do not directly describe the film morphology, were excluded (Figure S1). Table S1 summarizes the underlying rationale for excluding certain parameters from further consideration. The finalized set of parameters with their mutual correlations is provided in Figure S2. The finalized data set was divided into training and test sets at an 8:2 ratio for model construction and evaluation. The high- and low-performance class distributions are summarized in Table S2 for μ thresholds ranging from 0.2 to 2.0 cm^2^/(V·s) in increments of 0.2 cm^2^/(V·s). Prior to model training, all features were standardized using the mean and standard deviation of the training set to ensure consistent data scaling between the training and test sets. The primary analysis was conducted using this standard procedure. In addition, a supplementary grouping-based evaluation was performed to assess the potential effect of source level dependence among entries originating from the same publication or laboratory. The grouped split was selected to preserve the binary class distribution as closely as possible under this constraint.

### Machine Learning Model Optimization and Evaluation

Bayesian optimization^50^ was performed using the training set to determine the optimal hyperparameter values within the defined search space for the classification models (AdaBoost, CatBoost, Decision Tree, Extra Trees, Gradient Boosting, Histogram Gradient Boosting, LightGBM, Logistic, Multilayer Perception (MLP), Random Forest, Ridge, Stochastic Gradient Descent (SGD), Support Vector Machine (SVM), XGBoost). The detailed search space, cross-validation settings, and Bayesian search options are provided in Tables S3 and S4. Using the optimized hyperparameters, the bootstrap resampling method (2000 resamples) was applied to the test set to calculate the average receiver operating characteristic—area under the curve (ROC (receiver-operating characteristic curve)-AUC (area under the curve)) and standard deviation. The Brier score (mean squared error between predicted probabilities and observed outcomes) of the test set was also calculated as an additional metric for evaluating the model. In addition, the regression models (AdaBoost, CatBoost, Decision Tree, Elastic Net, Extra Trees, Gradient Boosting, Histogram Gradient Boosting, Lasso, LightGBM, MLP, Ordinary, Random Forest, Ridge, SGD, SVM, XGBoost) were evaluated to assess whether μ could be predicted directly from the parameters. The R^2^ value was used to evaluate the predictive performance of the regression models.

### Database Curation and Analysis

A detailed description of the data curation process, classification, and regression application is available via the GitHub link to the code provided in the Supporting Information, and the data set is available in the same GitHub repository.^51^

### ML-Based Parameter Prediction

To determine the key parameters that significantly affect the charge transport characteristics of DPP-DTT, as evaluated using OFETs, we employed a data-driven framework integrating ML modeling with experimental validation. This framework was designed to establish efficient experimental guidelines for OFET optimization by identifying the factors that significantly influence DPP-DTT performance in devices. Figure 1 presents three sequential steps in our framework: (*i*) data preparation, including data set extraction and preprocessing; (*ii*) ML modeling, including the identification of significant parameters and feature ranking; and (*iii*) experimentation, including OFET fabrication and investigation of feature effects. For classification, we defined high-performance and low-performance OFETs using a μ threshold of 1 cm^2^/V·s, which was selected as a practically meaningful benchmark based on prior reports in the field, including the representative μ of amorphous silicon and the saturated charge mobility reported for polythiophenes.^52,53^ Several ROC-AUC scores were calculated for multiple ML models, where the ROC curve represents the relationship between the true positive rate and false positive rate across different classification thresholds, and the AUC provides a threshold-independent summary of the ability of models to discriminate between different outcomes, with higher ROC-AUC values indicating stronger discrimination performance.^54^ In addition to ROC-AUC, the Brier score was used as an additional metric to further refine model selection by evaluating the accuracy of probabilistic predictions, with lower values indicating more accurate and better predictions.^55^ The model exhibiting the highest agreement with the experimental data was selected to evaluate the relative importance of each parameter. The selected parameter was subsequently used to guide experimental design.

**Figure 1:**
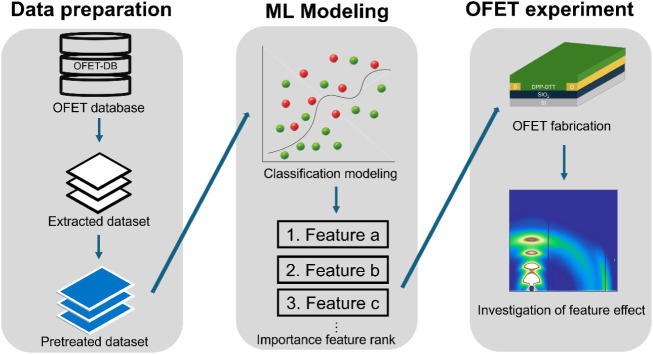
Workflow of the data-driven framework for identifying and validating principal process parameters associated with the performance of conjugated polymer-based OFETs.

### Materials and Device Characterization

#### Materials

DPP-DTT (*Mw* = 122 960 g/mol, PDI = 2.86, Ossila Ltd.), chloroform (CF; MilliporeSigma, HPLC, ≥99.8%, contains amylenes as stabilizer), o-dichlorobenzene (DCB; MilliporeSigma, anhydrous, 99%), chlorobenzene (CB; MilliporeSigma, anhydrous, 99.8%), toluene (TOL; MilliporeSigma, anhydrous, 99.8%), and *p*-xylene (XY; MilliporeSigma, anhydrous, ≥99%) were used without further purification.

### DPP-DTT Solution Preparation

DPP-DTT solutions (5 mg/mL) were prepared separately in CF, DCB, CB, TOL, and XY by adding the polymer and the corresponding solvent, to individual amber glass vials containing a magnetic stir bar. The mixtures were magnetically stirred on a hot plate as heating was initiated and then held at 40 °C for 12 h. At that time, heating was discontinued, and the vials containing the resulting solutions remained on the hot plate for 1 h with continuous stirring, after which they were at room temperature.

### OFET Fabrication and Characterization

Bottom-gate bottom-contact OFETs were fabricated using heavily p-doped silicon wafers purchased from Rogue Valley Microdevices. A 300 nm layer of thermally grown SiO_2_ on the wafer surface was used as the gate dielectric. Source and drain electrodes were deposited onto the SiO_2_ dielectric layer via standard photolithography liftoff techniques, followed by E-beam evaporation of a 3 nm Cr adhesion layer and 50 nm Au. The devices were then rinsed sequentially in acetone, methanol, and isopropyl alcohol and then dried under nitrogen flow. Subsequently, UV-ozone plasma treatment for 10 min was performed to remove organic contaminants on the device surface. The spin coating process was conducted at 1500 rpm for 2 min to deposit the polymer solutions onto the precleaned substrates under ambient conditions. The annealing process was carried out at 160 °C for 30 min under N_2_ atmosphere. Before OFET electrical properties were measured, the devices were stored in a nitrogen glovebox overnight. The charge mobility was calculated in the saturated regime from the transfer curve of drain current (I_D_) versus gate voltage (V_G_) using the following equation:

$$I_{D}=\frac{WC}{2L}\mu {\left(V_{G}-V_{T}\right)}^{2}$$

where W is channel width (2000 μm), L is channel length (50 μm), C is capacitance per unit area of the SiO_2_ dielectric layer (1.15 × 10^–8^ F·cm^–2^), μ is the hole mobility, and V_T_ is threshold voltage. The fit range is from V_G_ = −60 to −40 V. The output curves and transfer curves are provided in Figure S3 and Figure S4.

### Solution-State Structural Characterization

Solution-state wide-angle X-ray scattering (WAXS) measurements were performed at beamline 12-ID-E of the Advanced Photon Source (APS) at Argonne National Laboratory. Scattering profiles were collected using an incident X-ray beam with an energy of 13.3 keV and a sample-to-detector distance of 3622.78 mm. Polymer scattering data were corrected for background contributions determined from solvent-only measurements. Subsequent data analysis was conducted using SAXSLee, a MATLAB-based analysis tool.^56^

Cross-polarized optical microscopy (CPOM; Nikon Eclipse Ci-POL) solution-phase characterization was carried out by encapsulating polymer solutions between two glass coverslips, which were then placed on a Si wafer for reflection-mode CPOM measurements. High-concentration solutions were prepared using a drop-and-dry method, in which a controlled volume of the pristine solution was partially evaporated and the resulting residue was redissolved in 5 μL of solvent. The concentrated solution was then homogenized by shear alignment between the glass coverslips to further increase the effective concentration. Prior to imaging, the samples were subjected to two thermal annealing cycles to erase shear history and allow the system to reach an equilibrium state. Specifically, the temperature was first raised to near the boiling point of each solvent (CF: 50 °C, CB: 120 °C, DCB: 150 °C) and maintained for 10 min, then decreased at a controlled rate of 1 °C/min. Once the target temperature was reached, the samples were equilibrated for an additional 30 min before measurement.

### Solid-State Structural Characterization

#### UV–Vis–NIR Spectroscopy

Solid-state UV–vis–NIR spectra were obtained using an Agilent Cary 5000 spectrometer, which has a wavelength range of 175–3300 nm and a spectral bandwidth of 0.01–5 nm for UV–vis–NIR measurements. The corresponding measured DPP-DTT films were deposited onto a precleaned glass slide and annealed at 160 °C for 30 min for all spectral measurements.

#### Grazing Incidence Wide-Angle X-ray Scattering (GIWAXS)

GIWAXS measurements for CF, DCB, and CB samples were conducted at the 7.3.3 beamline of the Advanced Light Source (ALS) at Lawrence Berkeley National Laboratory, while those for TOL and XY samples were performed at the 9-ID-D beamline of the APS at Argonne National Laboratory, using incident angles of 0.119° and 0.12°, and X-ray energies of 10 and 11 keV, respectively. For sample preparation, the DPP-DTT solution was deposited onto a precleaned silicon wafer, followed by thermal annealing in the same way that OFET devices were fabricated. From GIWXAS measurements at ALS, the 2D scattering images were obtained and converted to 1D linecut profiles with the beamline NIKA package in Igor Pro.^57^ From GIWAXS measurements at APS, the 2D scattering images were obtained and converted to 1D linecut profiles with GIXSGUI.^58^

## Results and Discussion

The average ROC-AUC, standard deviation, and Brier score for the models explored here are provided in Table 1. The average ROC-AUC values with standard deviations and Brier scores for each model across different μ thresholds are summarized in Tables S5 and S6, respectively. Among the evaluated classifiers, Gradient Boosting exhibited a slightly higher average ROC-AUC, a relatively narrow standard deviation, and the lowest Brier score, which suggests that this classifier may be the most effective for further investigation of parameter importance. However, because the original train/test partitioning did not explicitly group samples by publication or laboratory, strict statistical independence of the test samples cannot be fully guaranteed. To address this point, an additional grouping-based evaluation was performed as a conservative robustness check using source-aware partitioning. Under this stricter evaluation framework, model performance estimates became more conservative, and the resulting feature importance pattern was less readily interpretable from a physical perspective, likely reflecting the limited size of the present data set (Tables S7–S10). This limitation may also have contributed to the tendency toward higher model performance at higher μ thresholds, where the number of high-performance devices progressively decreased (Tables S5–S6). For the regression models, the average R^2^ values for both the training and test sets were generally low, whereas the corresponding standard deviations were relatively high (Table S11). This suggests that direct prediction of μ from the parameters may not be sufficiently reliable. Therefore, the Gradient Boosting-based feature ranking is interpreted here as a practical guide for subsequent experimental validation rather than as a definitive mechanistic conclusion derived solely from the model output. When Gradient Boosting was used to evaluate feature importance, the identified important parameters were in order of *M*_w_, PDI, MGC, E_T_^N^, solution concentration, SAM treatment, insulating polymer blending, annealing, post chemical processing, and poor solvent addition. This ranked order was quantified by permutation importance analysis (Table S12). Among the identified features, it has been reported that controlling the PDI is experimentally challenging.^59^ Further, with the exception of E_T_^N^, the remaining features have already been shown to influence conjugated polymer solid state morphology, charge carrier transport characteristics, and OFET performance.^7,26,37,39,60–62^

**Table 1:** Performance Metrics Including Average ROC-AUC, Standard Deviation, and Brier Score for Each ML Classifier

Classifier Model	Average ROC-AUC	Standard Deviation	Brier Score
Gradient Boosting	0.9977	0.0038	0.0168
CatBoost	0.9977	0.0038	0.0177
Extra Trees	0.9977	0.0038	0.0265
Random Forest	0.9935	0.0088	0.0202
SVM	0.9935	0.0088	0.0378
SGD	0.9935	0.0088	0.0577
Ridge	0.9935	0.0088	0.1400
Hist Gradient Boosting	0.9932	0.0094	0.0316
XGBoost	0.9932	0.0094	0.0316
LightGBM	0.9932	0.0094	0.0383
AdaBoost	0.9932	0.0094	0.0398
Logistic	0.9926	0.0099	0.0289
MLP	0.9802	0.0189	0.0453
Decision Tree	0.9100	0.0905	0.0395

Based on the above data-driven approach, among the identified parameters, E_T_^N^ emerged, for the first time, as a quantified descriptor for solvent choice, as a potentially significant factor influencing DPP-DTT (Figure 2a) assembly into structures that support enhanced charge carrier transport that underlies device performance. In the present data set, E_T_^N^ was also correlated with several other solvent descriptors and is interpreted here as a representative empirical descriptor of broader solvent-dependent effects. To investigate how E_T_^N^ influences DPP-DTT charge transport performance, solvents capable of dissolving DPP-DTT were examined first (Table S13). Among the screened solvents, five—XY, TOL, CB, DCB, and CF—were selected for further study based on solubility. With these selected solvents, bottom-gate bottom-contact OFETs were fabricated by spin coating the corresponding DPP-DTT solutions onto the as-prepared substrates. The μ, and threshold voltage (V_T_) as a function of E_T_^N^ are presented in Figure 2b. Plots showing the relationships between solvent descriptors excluded from the data set due to multicollinearity and OFET performance are provided in the Supporting Information (Figures S5–S7). The similar μ trends observed with the Hansen solubility parameters, particularly polarity and hydrogen bonding, as well as Henry’s law constant, likely reflect their correlation with E_T_^N^. Accordingly, these descriptors are not interpreted as independent competing variables, but as reflecting related features of the same overall solvent effect. As shown in Figure 2b, the μ exhibits a clear increasing trend with increasing E_T_^N^. Devices processed from the two solvents with the lowest E_T_^N^ values, XY (0.041 cm^2^/(V·s)) and TOL (0.039 cm^2^/(V·s)), exhibit nearly identical mobility values. A noticeable improvement is observed for devices prepared from CB (0.100 cm^2^/(V·s)), followed by a further increase in DCB (0.158 cm^2^/(V·s)). The highest mobility is obtained from devices fabricated using CF (0.239 cm^2^/(V·s)), which has the highest E_T_^N^ value among the solvents identified here. On the other hand, V_T_ exhibits the opposite trend to μ. Devices processed from XY exhibit a positive V_T_ value (12.711 V), which may be related to charge trapping.^63^ In contrast, devices processed from TOL (−14.147 V), CB (−16.106 V), DCB (−23.776 V), and CF (−22.932 V) display native V_T_ values. No clear trend was observed I_on_/_off_ vs E_T_^N^ (Figure S7a). The noticeable variability in I_on_/_off_ for the device processed from TOL may be associated with increased off-current leakage.^64^

**Figure 2:**
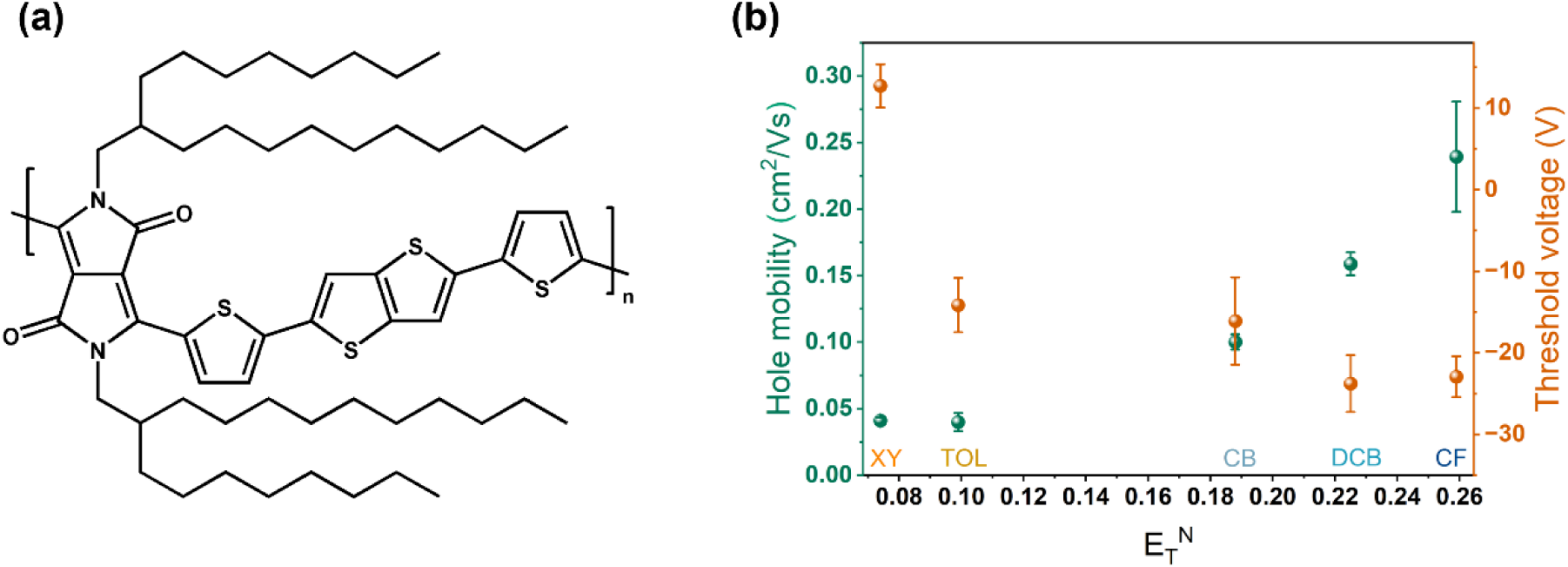
(a) Structural representation of DPP-DTT, (b) hole mobility, and threshold voltage of DPP-DTT OFETs as a function of E_T_^N^. Error bars here represent the standard deviation obtained from 8 to 12 OFET devices.

### Unveiling Film Structure through Optical and Structural Analysis

By correlating DPP-DTT thin-film UV–vis–NIR absorption characteristics with X-ray diffraction results, significant decoupling between short-range excitonic interactions, as probed by UV–vis–NIR absorption, and long-range structural order revealed by GIWAXS were uncovered. These findings identify the key thin-film morphological/structural features responsible for the enhanced performance observed in the case of high E_T_^N^ solvents.

Figure 3a presents the UV–vis–NIR absorption spectra of the 5 mg/mL precursor solutions (dashed lines) with the corresponding solidified thin-films (solid lines). Most notably, all films exhibit spectral signatures consistent with H-aggregate formation, a conclusion supported by two key observations. First, the solidified films display a blue shift of the main absorption band relative to the solution state, consistent with the Kasha exciton model, in which side-by-side π-stacking of polymer chains (J_0_ > 0) raises the energy of the optically allowed (k = 0) exciton.^65^ Second, the vibronic progression shows a suppressed 0–0 transition, with R_abs_ (A_(0–0)_/A_(0–1)_) < 1, as predicted by the Spano model for strong interchain excitonic coupling (W).^65,66^ Therefore, the combined observation of a blue-shifted maximum and reduced R_abs_ provides evidence for enhanced H-aggregation upon film formation.

**Figure 3:**
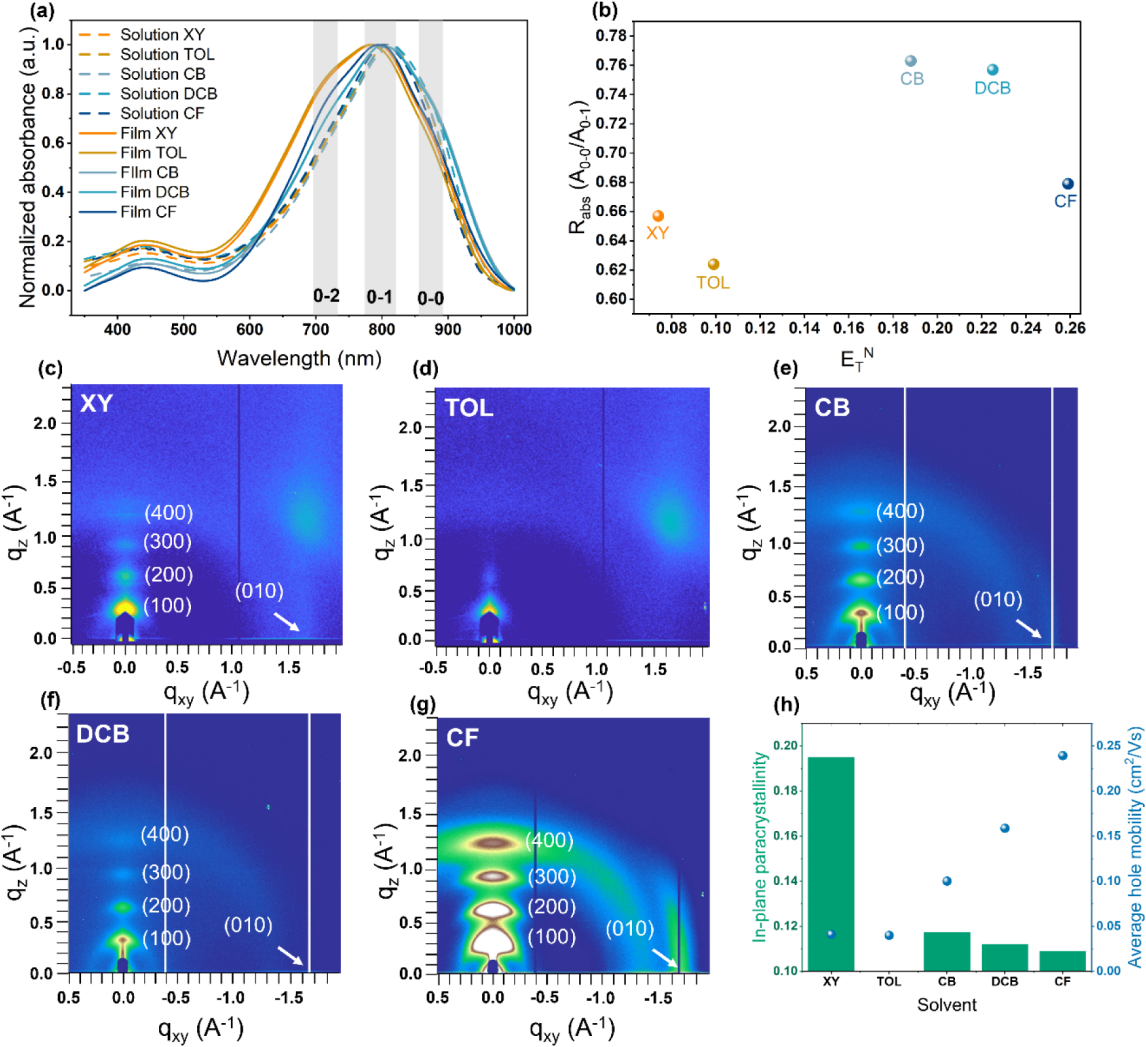
(a) Normalized UV–vis–NIR absorption spectra of DPP-DTT in solution and in thin films processed from different solvents, (b) vibronic intensity ratio R_abs_(= A_(0–0)_/A_(0–1)_) extracted from the film spectra, GIWAXS scattering of DPP-DTT films from (c) XY, (d) TOL, (e) DCB, (f) CB, and (g) CF, and (h) DPP-DTT in-plane paracrystallinity and hole mobility as a function of processing solvent.

While the solution-state spectra show negligible variation across solvents, the solid-state absorption profiles exhibit significant solvent-dependent differences. The Spano photophysical framework was utilized to estimate the interchain excitonic coupling strength within each film.^65^ In this model, the R_abs_ ratio for H-aggregates is inversely proportional to the coupling strength (W); as interchain interactions intensify, oscillator strength is progressively transferred from the A_(0–0)_ transition into the higher-energy vibronic manifold (e.g., A_(0–1)_, A_(0–2)_). Thus, variations in R_abs_ across films serve as a direct proxy for the degree of short-range excitonic coupling; samples exhibiting the lowest R_abs_ values therefore possess the strongest interchain excitonic interactions. Following the model, R_abs_ values for films processed from different solvents were quantified (Figure 3b). Films cast from XY and TOL exhibited the lowest R_abs_ values (indicating the strongest coupling), while those processed from CB and DCB showed the highest. CF films displayed intermediate values. In summary, UV–vis–NIR analysis confirms that while all films form H-aggregates, the coupling strength varies nonmonotonically with E_T_^N^: solvents with low E_T_^N^ yield strong coupling, intermediate E_T_^N^ solvents yield weak coupling, and the highest E_T_^N^ solvent (CF) returns to an intermediate coupling regime.

Even though the analysis of local coupling strength elucidates interactions at the molecular scale, it does not fully capture macroscopic film properties which are critical for charge transport. To address this discrepancy, we employed GIWAXS to correlate the charge carrier mobility with long-range structural characteristics of the bulk films. The resulting 2D diffraction patterns are presented in Figure 3c–g, while the corresponding 1D line cuts and detailed peak fitting procedures, including peak deconvolution, are provided in Figures S8–S9. Peak positions used for fitting were assigned with previously reported values,^61^ and all extracted structural parameters are summarized in Table S14.

A distinct solvent dependence is immediately apparent from the GIWAXS data. Films processed from XY exhibited in-plane (IP) π–π stacking and out-of-plane (OOP) lamellar scattering, whereas films processed from TOL showed negligible scattering features. Compared to the films processed from high E_T_^N^ solvents, these features were poorly defined and low in intensity, indicating a globally low degree of crystallinity and weak long-range molecular packing. In contrast, films processed from CB, DCB, and CF all displayed clear diffraction peaks corresponding to OOP lamellar stacking, as well as both IP and OOP π-π stacking peaks. Based on the 2D GIWAXS scattering, all films except for the film processed from TOL were interpreted as predominantly edge on. Notably, these films also exhibited a broader scattering ring located at q = 1.251 Å^–1^ (CB), 1.273 Å^–1^ (DCB), and 1.250 Å^–1^ (CF), attributable to the LC phase, indicative of mesoscopic ordering associated with LC-mediated assembly. This signature suggests the presence of a lyotropic LC-mediated assembly pathway during DPP-DTT thin-film fabrication—a mechanism previously reported to facilitate enhanced crystallinity and superior structural order in conjugated polymer films.^28,67,68^

Quantitative analysis of the scattering data bridges the gap between the local molecular interactions inferred from photophysical analysis and the macroscopic structural properties that govern charge carrier transport. The molecular packing distances were examined first by peak fitting the 1D line-cuts to investigate the small-scale interactions identified in the optical analysis. The structural parameters are provided in Table 2. For films processed from XY, we observed the smallest π-π stacking distance (0.374 nm). In contrast, films cast from CB and DCB exhibited the largest π-π stacking distance (0.381 nm), while CF displayed intermediate values. This structural trend directly corroborates the UV–vis–NIR findings (*vide supra*) regarding excitonic coupling strength; the tighter molecular packing observed in XY aligns with the strong interchain coupling, whereas the expanded lattice in CB and DCB corresponds to the weaker coupling regime.

**Table 2:** Structural Parameters Extracted from GIWAXS Analysis of DPP-DTT films^a^

Sample	Lamellar (nm)	In-plane d_π–π_ (nm)	LC phase (nm)	In-plane g
XY	2.159	0.374	-	0.195
TOL	-	-	-	-
CB	1.950	0.381	5.024	0.118
DCB	1.963	0.381	4.933	0.112
CF	1.933	0.375	5.027	0.108

a g: Paracrystallinity.

Subsequently, parameters related to long-range molecular order, specifically focusing on IP paracrystallinity are key determinants of carrier mobility.^69,70^ Interestingly, a clear correlation with solvent polarity was observed: as solvent E_T_^N^ increases, paracrystallinity decreases. This trend mirrors μ measurements, confirming that long-range structural integrity is the dominant factor driving charge transport performance.

In sum, films processed from TOL and XY exhibit the strongest local excitonic coupling but suffer from poor crystallinity, resulting in low device performance. Conversely, CB and DCB produce films with weaker local excitonic coupling yet demonstrate well-defined lamellar and π–π stacking with moderate paracrystallinity, reflecting improved long-range order. Most notably, CF yields films that combine relatively strong excitonic coupling with shorter π–π stacking distances than CB or DCB, and the highest overall crystallinity. This optimized morphology directly correlates with the superior charge transport performance observed in the CF-processed devices.

### Unraveling the Solution-State Origins of Film Morphology

While the solid-state analysis successfully establishes a correlation between macroscopic film morphology and charge carrier transport characteristics, several critical questions remain unresolved. First, the apparent decoupling between short-range excitonic coupling and long-range structural order warrants further explanation; specifically, what underlying driving forces create this discrepancy. Second, from a mechanistic perspective, why does use of a high E_T_^N^ solvent, such as CF, result in the most optimized morphology and superior device performance? To elucidate the mechanistic origins of the observed solvent-dependent morphologies, the solution-state behavior of DPP-DTT was investigated, specifically focusing on the preaggregation and self-assembly processes that preceded thin-film formation.

First, the aggregation state of each polymer solution was evaluated using transmission optical microscopy (T-OM) to probe micron-scale features; and solution-state WAXS enabled analysis of molecular-scale interactions. XY and TOL solutions, characterized by low E_T_^N^, appeared cloudy and turbid, revealing macroscopic aggregates under T-OM. Correspondingly, solution-state WAXS profiles exhibited a pronounced π–π stacking peak at 4.16 Å, a definitive signature of solution aggregation (Figure 4a,f and Figure 4b,g). In stark contrast, CB and DCB solutions were optically clear and featureless; their WAXS profiles showed no discernible scattering peaks, indicating absence of backbone aggregates (Figure 4c,h and Figure 4d,i). CF occupied a unique intermediate regime: while the solutions were optically clear, WAXS revealed a weak, yet distinct π–π stacking peak at 4.19 Å, suggesting the presence of limited but definitive backbone aggregation (Figure 4e, j).

**Figure 4:**
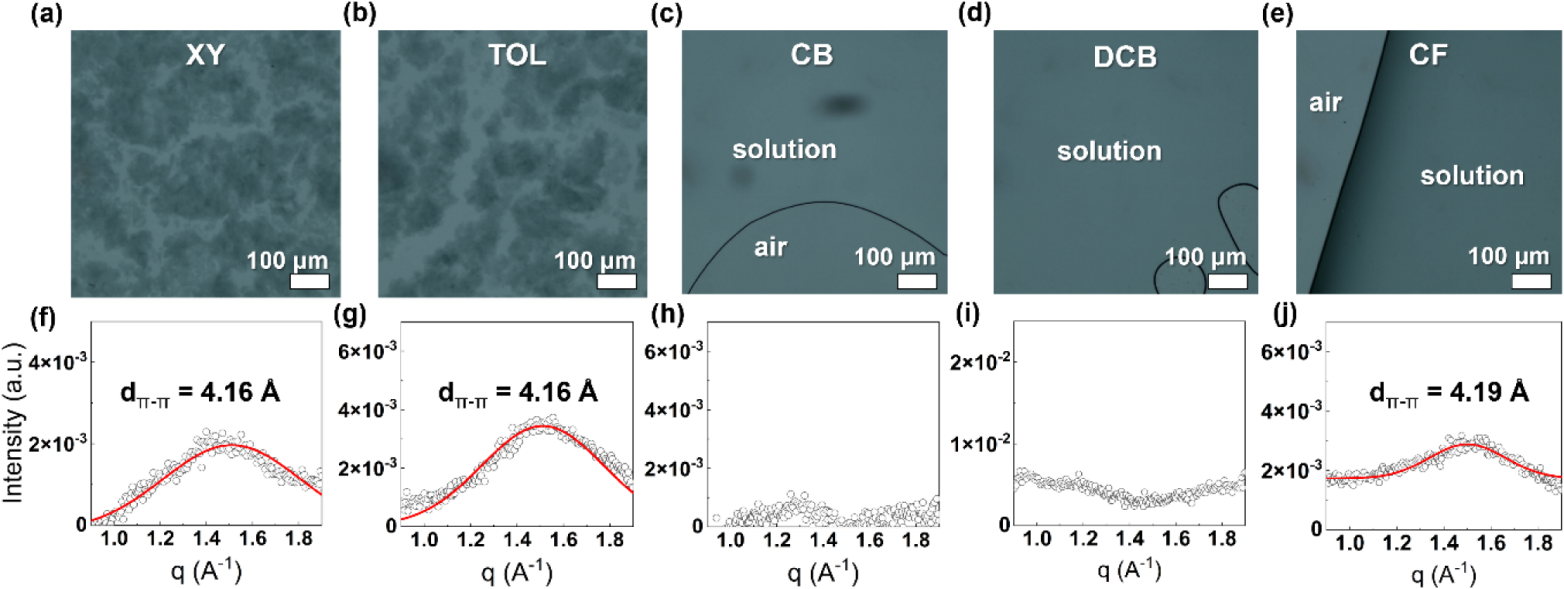
Transmission optical microscopy images of DPP-DTT solutions prepared in (a) XY, (b) TOL, (c) CB, (d) DCB, and (e) CF at a concentration of 5 mg/mL. Corresponding solution-state wide-angle X-ray scattering analyses are shown for (f) XY, (g) TOL, (h) CB, (i) DCB, and (j) CF.

These solution-state observations provide a link to the solid-state optical properties discussed above. The substantial DPP-DTT aggregation in TOL and XY, a manifestation of poor solvent quality, directly correlates with the observed strong interchain excitonic coupling in the respective solidified films.^71^ Excessive aggregation in TOL and XY also explains the detrimental structural outcomes. We hypothesize that these large aggregates prevent liquid crystal formation during solvent evaporation, and thus limit macroscopic long-range order, resulting in poor charge carrier transport. Conversely, the high solubility offered by CB and DCB delay backbone aggregation needed for liquid crystal formation during solvent evaporation. Upon rapid solvent evaporation, the chains become kinetically trapped in a disordered configuration, explaining the observed weak excitonic coupling. CF occupies an intermediate regime, showing the ideal extent of aggregation to best facilitate liquid crystal phase formation.^72^

To validate the hypothesis proposed above, we studied the concentration-dependent assembly pathways during solvent drying. While solution aggregation successfully accounts for the observed trends in local excitonic coupling from UV–vis–NIR spectroscopy, it does not fully explain why CF produces superior long-range order compared to CB and DCB, particularly given its intermediate coupling strength. To resolve this apparent paradox, we investigated the solvent-dependent assembly pathways using cross-polarized optical microscopy (CPOM). Specifically, we investigated the equilibrium states of the polymer across a range of concentrations to elucidate the assembly pathway during solvent evaporation.

In CF, increasing the concentration of DPP-DTT to 30, 50, 100, and 200 mg/mL led to the progressive growth and evolution of LC domains (Figure 5a–d). At 30 mg/mL, small, submicron homogeneous tactoids were observed, while increasing the concentration to 50 mg/mL, these structures evolved into well-defined bipolar tactoids with sizes ranging from several to tens of micrometers. Above 100 mg/mL, extended nematic LC phases emerged. Both CB (Figure 5e–h) and DCB (Figure 5i–l) solutions exhibited qualitatively similar LC-mediated assembly behavior; however, the onset of LC ordering occurred at substantially higher concentrations. In these cases, no birefringence was observed below 100 mg/mL, with LC tactoids appearing only at 150 mg/mL, and nematic phases forming at DPP-DTT concentrations exceeding 200 mg/mL. The delayed LC formation is attributed to stronger solvation and reduced aggregation of DPP-DTT in CB and DCB, in contrast to CF, where aggregated species facilitate LC-mediated assembly.

**Figure 5:**
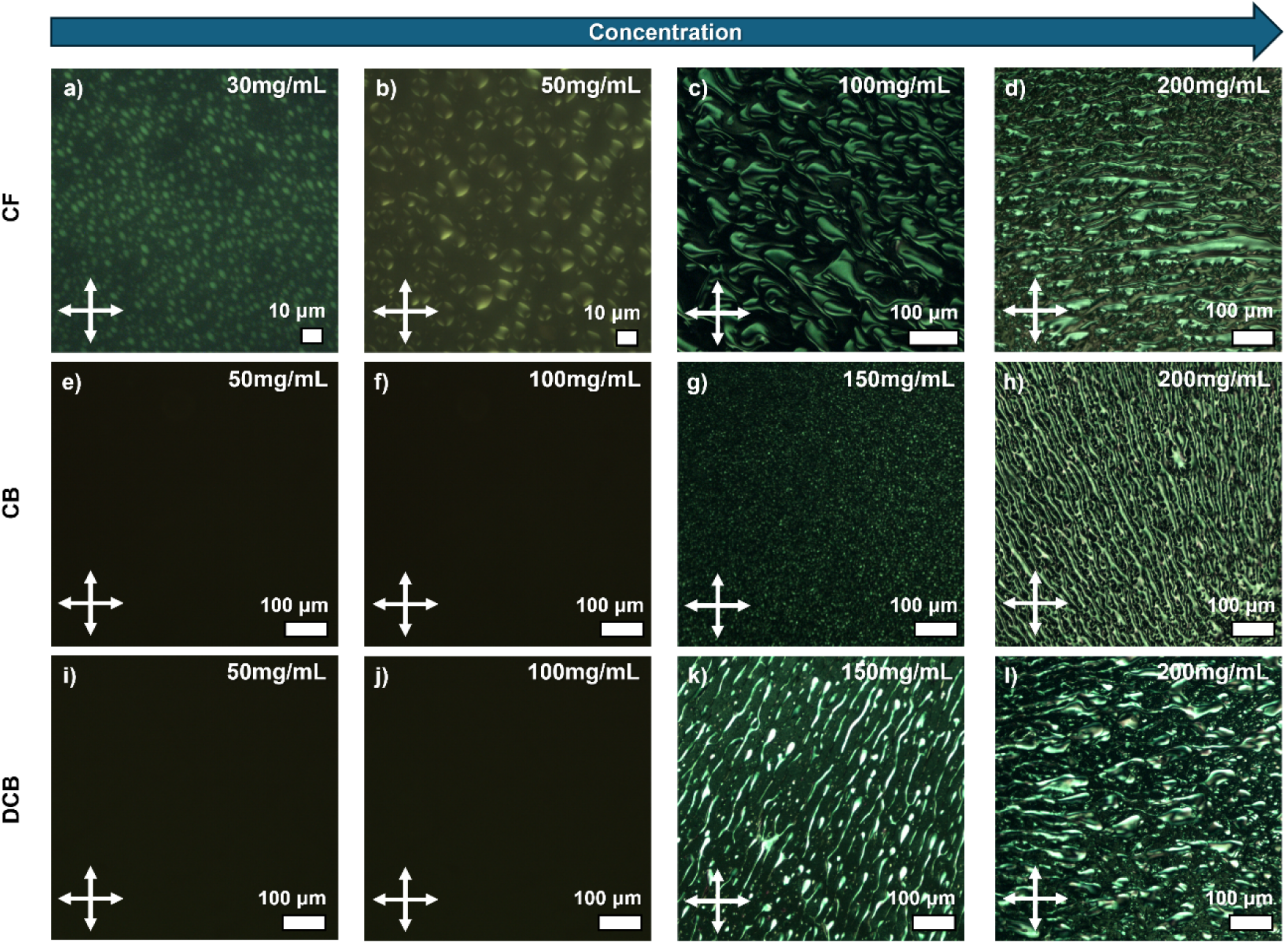
Investigation of the solvent-dependent assembly pathways of DPP–DTT in CF, CB, and DCB. Cross-polarized optical microscopy (CPOM) images reveal the assembly behavior of DPP–DTT solutions prepared in (a–d) CF, (e–h) CB, and (i–l) DCB. All solutions exhibit liquid-crystal (LC)–mediated assembly pathways, with LC features becoming increasingly apparent under CPOM as the concentration increases. Notably, CF induces LC ordering at relatively low concentrations (30 mg/mL), whereas CB and DCB require significantly higher concentrations (150 mg/mL) to form LC phases.

The results confirm that CF, characterized by its high E_T_^N^, facilitates an LC-mediated assembly pathway. This mechanism is well-established for its ability to enhance long-range order and crystallinity, thereby explaining the superior structural quality and device performance of CF-processed films relative to those cast from CB or DCB. Crucially, this finding resolves the apparent paradox of how CF films achieve exceptional long-range order and charge transport despite exhibiting only intermediate local excitonic coupling. Unlike CB and DCB, which require high concentrations to access LC phases and are therefore prone to kinetically trapped, disordered morphologies, CF may enable a facile LC-mediated templating process that directs polymer chains into highly aligned, crystalline domains during solvent evaporation. This result suggests that the choice of solvent significantly influences the kinetics of reaching the LC phase, a process in which kinetic factors are well-established to play a dominant role.^73,74^ Importantly, the tendency toward LC ordering in CF is already evident in the concentration-dependent equilibrium states before film formation, indicating that this behavior cannot be attributed to its lower boiling point. Differences in evaporation kinetics during film deposition may also contribute to the assembly pathway. Further clarification of this effect would require additional *in situ* characterization beyond the scope of the present study.

While a comprehensive elucidation of the origins of different kinetics in LC formation depending on the solvent lies beyond the scope of the current study, we speculate that it arises from two synergistic factors. First, high E_T_^N^ solvents like CF may promote side-chain-assisted aggregation, a process conducive to LC ordering, possibly because CF can hydrogen bond with the conjugated backbone. Second, the specific solvation environment in CF likely allows for partial backbone solvation, providing sufficient mobility for self-organization while preserving the critical interchain interactions required for LC phase formation.

## Conclusions

Using a data science approach, in this study we identified solvent polarity as a critical parameter that plays a significant role in the solution-to-film assembly pathway of DPP-DTT. By integrating data-driven estimation with experimental validation, we found that solvents with higher E_T_^N^ values promote an LC-mediated polymer assembly pathway, as revealed by CPOM observations. This pathway leads to improved long-range structural order, as confirmed by GIWAXS, which in turn explains the enhanced charge transport characteristics as evaluated via OFET device-derived μ measurements. The results demonstrate an effective approach for uncovering key parameters to be considered in the design of robust process protocols for the solution state deposition of thin polymer films from complex polymer systems. Further, the study provides a framework for establishing data-guided experimental strategies for solution-processed organic electronics.

## Supplementary Material



## Notes

Additional details on data set preprocessing, machine learning optimization and validation, DPP-DTT solubility tests, solvent parameter-dependent OFET characteristics, supplementary GIWAXS analysis, output and transfer curves, classifier performance metrics, and permutation importance analyses (PDF)

## References

[1] Yu N., Zhuo Z., Zheng Y., Ni M., Chen W., Liu J., He D., Ma J., Xu M., An X., Bai L., Lin J., Wang Z., Huang W. (2025). Hierarchically Plasticized
Intrinsically Stretchable Fully π-Conjugated Polymer Films for
Flexible and Large-Area Printed RGB Light-Emitting Diodes. Chem. Eng. J..

[2] Jordan R. S., Wang Y. (2019). 3D Printing of Conjugated Polymers. J. Polym.
Sci., Part B: Polym. Phys..

[3] Ashizawa M., Zheng Y., Tran H., Bao Z. (2020). Intrinsically Stretchable
Conjugated Polymer Semiconductors in Field Effect Transistors. Prog. Polym. Sci..

[4] Yang J., Zhao Z., Wang S., Guo Y., Liu Y. (2018). Insight into
High-Performance Conjugated Polymers for Organic Field-Effect Transistors. Chem..

[5] Yan Y., Duan B., Ru M., Gu Q., Li S., Zhao W. (2025). Toward Flexible and Stretchable Organic Solar Cells: A Comprehensive
Review of Transparent Conductive Electrodes, Photoactive Materials,
and Device Performance. Adv. Energy Mater..

[6] Li M., Hua L., Liu J., Ren Z. (2023). Recent Advances in Regulating the
Excited States of Conjugated Thermally Activated Delayed Fluorescence
Polymers for High-Efficiency OLEDs. Mater. Chem.
Front..

[7] Tran V. V., Jeong G., Wi E., Lee D., Chang M. (2023). Design and
Fabrication of Ultrathin Nanoporous Donor-Acceptor Copolymer-Based
Organic Field-Effect Transistors for Enhanced VOC Sensing Performance. ACS Appl. Mater. Interfaces.

[8] Dong H., Hu W. (2016). Multilevel Investigation
of Charge Transport in Conjugated Polymers. Acc. Chem. Res..

[9] Liu Z., Zhang G., Zhang D. (2018). Modification
of Side Chains of Conjugated
Molecules and Polymers for Charge Mobility Enhancement and Sensing
Functionality. Acc. Chem. Res..

[10] Wu Y., Zhao Y., Liu Y. (2021). Toward Efficient Charge Transport
of Polymer-Based Organic Field-Effect Transistors: Molecular Design,
Processing, and Functional Utilization. Acc.
Mater. Res..

[11] Li W., Yang L., Tumbleston J. R., Yan L., Ade H., You W. (2014). Controlling Molecular Weight of a High Efficiency Donor-Acceptor
Conjugated Polymer and Understanding its Significant Impact on Photovoltaic
Properties. Adv. Mater..

[12] Pei D., Wang Z., Peng Z., Zhang J., Deng Y., Han Y., Ye L., Geng Y. (2020). Impact of Molecular Weight on the
Mechanical and Electrical Properties of a High-Mobility Diketopyrrolopyrrole-Based
Conjugated Polymer. Macromolecules.

[13] Zheng Y. Q., Yao Z. F., Lei T., Dou J. H., Yang C. Y., Zou L., Meng X., Ma W., Wang J. Y., Pei J. (2017). Unraveling
the Solution-State Supramolecular Structures of Donor-Acceptor Polymers
and their Influence on Solid-State Morphology and Charge-Transport
Properties. Adv. Mater..

[14] Sun M., Sun Z., Zheng Y., Kim R., Liu A. L., Richter L. J., Gilchrist J. F., Reichmanis E. (2025). Preprocessing Affords 3D Crystalline
Poly­(3-hexylthiophene) Structure. Chem. Mater..

[15] Khim D., Baeg K. J., Kim J., Kang M., Lee S. H., Chen Z., Facchetti A., Kim D. Y., Noh Y. Y. (2013). High Performance
and Stable N-Channel Organic Field-Effect Transistors by Patterned
Solvent-Vapor Annealing. ACS Appl. Mater. Interfaces.

[16] Callaway C.
P., Liu A. L., Venkatesh R., Zheng Y., Lee M., Meredith J. C., Grover M., Risko C., Reichmanis E. (2022). The Solution
is the Solution: Data-Driven Elucidation of Solution-to-Device Feature
Transfer for pi-Conjugated Polymer Semiconductors. ACS Appl. Mater. Interfaces.

[17] Khasbaatar A., Jones A. L., Fernando P. S., Sai H., Zhu C., Gann E., Reynolds J. R., Diao Y. (2024). Tuning the
Solution
Aggregate Structure of a PM7-Based Conjugated Polymer to Enable Green
Solvent Processing of Organic Solar Cells. Chem.
Mater..

[18] Wang Z.-Y., Di Virgilio L., Yao Z.-F., Yu Z. D., Wang X.-Y., Zhou Y.-Y., Li Q.-Y., Lu Y., Zou L., Wang H. (2021). Correlating Charge Transport Properties of
Conjugated
Polymers in Solution Aggregates and Thin-Film Aggregates. Angew. Chem., Int. Ed..

[19] Yao Z.-F., Wang J.-Y., Pei J. (2023). Controlling Morphology and Microstructure
of Conjugated Polymers via Solution-State Aggregation. Prog. Polym. Sci..

[20] Zheng Y.-Q., Yao Z.-F., Dou J.-H., Wang Y., Ma W., Zou L., Nikzad S., Li Q.-Y., Sun Z.-H., Yu Z.-A., Zhang W.-B., Wang J.-Y., Pei J. (2021). Influence of Solution-State
Aggregation on Conjugated Polymer Crystallization in Thin Films and
Microwire Crystals. Giant.

[21] Rosas
Villalva D., Derewjanko D., Zhang Y., Liu Y., Bates A., Sharma A., Han J., Gibert-Roca M., Arteaga O. Z., Jang S., Moro S., Costantini G., Gu X., Kemerink M., Baran D. (2025). Intermolecular-Force-Driven Anisotropy
Breaks the Thermoelectric Trade-Off in n-Type Conjugated Polymers. Nat. Mater..

[22] Xu Z., Park K. S., Diao Y. (2020). What Is the
Assembly Pathway of a
Conjugated Polymer From Solution to Thin Films?. Front. Chem..

[23] Jiang Y., Yao C., Yang Y., Wang J. (2024). Machine Learning Approaches for Predicting
Power Conversion Efficiency in Organic Solar Cells: A Comprehensive
Review. Sol. RRL.

[24] Liu A. L., Lee M., Venkatesh R., Bonsu J. A., Volkovinsky R., Meredith J. C., Reichmanis E., Grover M. A. (2023). Conjugated Polymer
Process Ontology and Experimental Data Repository for Organic Field-Effect
Transistors. Chem. Mater..

[25] Alesadi A., Cao Z., Li Z., Zhang S., Zhao H., Gu X., Xia W. (2022). Machine Learning
Prediction of Glass Transition Temperature of Conjugated
Polymers from Chemical Structure. Cell Rep.
Phys. Sci..

[26] Venkatesh R., Zheng Y., Viersen C., Liu A., Silva C., Grover M., Reichmanis E. (2021). Data Science Guided Experiments Identify
Conjugated Polymer Solution Concentration as a Key Parameter in Device
Performance. ACS Mater. Lett..

[27] Xu Z., Tsai H., Wang H.-L., Cotlet M. (2010). Solvent Polarity Effect
on Chain Conformation, Film Morphology, and Optical Properties of
a Water-Soluble Conjugated Polymer. J. Phys.
Chem. B.

[28] Khasbaatar A., Damron A. M., Fernando P. S., Williams J. S., Zhu C., Gann E. H., Lee J. H., Birge A., Kim B., Sabury S., Lee M. L., Reynolds J. R., Diao Y. (2025). Lyotropic
Liquid Crystal Mediated Assembly of Donor Polymers Enhances Efficiency
and Stability of Blade-Coated Organic Solar Cells. Adv. Mater..

[29] Reichardt C. (2007). Solvents and
Solvent Effects: An Introduction. Org. Process
Res. Dev..

[30] Pandian R., Burda H., Alfurayj I., Reichardt C., Burda C. (2024). 60 Years of Betaine 30 horizontal line From Solvatochromic Discovery
to Future Frontiers. J. Phys. Chem. B.

[31] Spange S., Weiß N., Schmidt C. H., Schreiter K. (2020). Reappraisal
of Empirical Solvent Polarity Scales for Organic Solvents. Chem.–Methods.

[32] Ranjkesh A., Parast M. H., Strzezysz O., Zakerhamidi M. S., Yoon T.-H. (2018). New Linear Solvation Energy Relationships for Empirical
Solvent Scales Using the Kamlet-Abboud-Taft Parameter Sets in Nematic
Liquid Crystals. RSC Adv..

[33] Spange S., Lienert C., Friebe N., Schreiter K. (2020). Complementary
Interpretation of E­(T)(30). Polarity Parameters of Ionic Liquids. Phys. Chem. Chem. Phys..

[34] Lei Y., Wu B., Chan W.-K. E., Zhu F., Ong B. S. (2015). Engineering Gate
Dielectric Surface Properties for Enhanced Polymer Field-Effect Transistor
Performance. J. Mater. Chem. C.

[35] Zhang G., McBride M., Persson N., Lee S., Dunn T. J., Toney M. F., Yuan Z., Kwon Y.-H., Chu P.-H., Risteen B., Reichmanis E. (2017). Versatile
Interpenetrating Polymer
Network Approach to Robust Stretchable Electronic Devices. Chem. Mater..

[36] Zhang G., Lee S., Gutiérrez-Meza E., Buckley C., McBride M., Valverde-Chávez D. A., Kwon Y. H., Savikhin V., Xiong H., Dunn T. J., Toney M. F., Yuan Z., Silva C., Reichmanis E. (2019). Robust and
Stretchable Polymer Semiconducting
Networks: From Film Microstructure to Macroscopic Device Performance. Chem. Mater..

[37] Li J., Zhao Y., Tan H. S., Guo Y., Di C.-A., Yu G., Liu Y., Lin M., Lim S. H., Zhou Y., Su H., Ong B. S. (2012). A Stable
Solution-Processed Polymer Semiconductor with
Record High-Mobility for Printed Transistors. Sci. Rep..

[38] Lei Y., Deng P., Li J., Lin M., Zhu F., Ng T. W., Lee C. S., Ong B. S. (2016). Solution-Processed
Donor-Acceptor Polymer Nanowire Network Semiconductors For High-Performance
Field-Effect Transistors. Sci. Rep..

[39] Afzal T., Iqbal M. J., Iqbal M. Z., Sajjad A., Raza M. A., Riaz S., Kamran M. A., Numan A., Naseem S. (2020). Effect of
Post-Deposition Annealing Temperature on the Charge Carrier Mobility
and Morphology of DPPDTT-Based Organic Field-Effect Transistors. Chem. Phys. Lett..

[40] Sarkar T., Schneider S. A., Ankonina G., Hendsbee A. D., Li Y., Toney M. F., Frey G. L. (2020). Tuning Intra and Intermolecular Interactions
for Balanced Hole and Electron Transport in Semiconducting Polymers. Chem. Mater..

[41] Iqbal M. J., Haq H., Riaz S., Raza M. A., Iqbal M. Z., Chaudhry M. U., Naseem S. (2019). On the Operational,
Shelf Life and Degradation Mechanism
in Polymer Field-Effect Transistors. Superlattices
Microstruct..

[42] Xi Y., Wolf C. M., Pozzo L. D. (2019). Self-Assembly of Donor-Acceptor Conjugated
Polymers Induced by Miscible ‘Poor’ Solvents. Soft Matter.

[43] Kafle P., Zhang F., Schorr N. B., Huang K. Y., Rodríguez-López J., Diao Y. (2020). Printing 2D Conjugated Polymer Monolayers and Their Distinct Electronic
Properties. Adv. Funct. Mater..

[44] Armin A., Wolfer P., Shaw P. E., Hambsch M., Maasoumi F., Ullah M., Gann E., McNeill C. R., Li J., Shi Z., Burn P. L., Meredith P. (2015). Simultaneous Enhancement of Charge
Generation Quantum Yield and Carrier Transport in Organic Solar Cells. J. Mater. Chem. C.

[45] Qu G., Zhao X., Newbloom G. M., Zhang F., Mohammadi E., Strzalka J. W., Pozzo L. D., Mei J., Diao Y. (2017). Understanding
Interfacial Alignment in Solution Coated Conjugated Polymer Thin Films. ACS Appl. Mater. Interfaces.

[46] Luo H., Yu C., Liu Z., Zhang G., Geng H., Yi Y., Broch K., Hu Y., Sadhanala A., Jiang L., Qi P., Cai Z., Sirringhaus H., Zhang D. (2016). Remarkable Enhancement of Charge
Carrier Mobility of Conjugated Polymer
Field-Effect Transistors upon Incorporating an Ionic Additive. Sci. Adv..

[47] Chen Z., Lee M. J., Ashraf R. S., Gu Y., Albert-Seifried S., Nielsen M. M., Schroeder B., Anthopoulos T. D., Heeney M., McCulloch I. (2012). High-Performance Ambipolar
Diketopyrrolopyrrole-Thieno­[3,2-b]­thiophene Copolymer Field-Effect
Transistors with Balanced Hole and Electron Mobilities. Adv. Mater..

[48] Mohammadi E., Zhao C., Meng Y., Qu G., Zhang F., Zhao X., Mei J., Zuo J.-M., Shukla D., Diao Y. (2017). Dynamic-Template-Directed Multiscale Assembly for Large-Area Coating
of Highly-Aligned Conjugated Polymer Thin Films. Nat. Commun..

[49] Lei Y., Deng P., Zhang Q., Xiong Z., Li Q., Mai J., Lu X., Zhu X., Ong B. S. (2018). Hydrocarbons-Driven
Crystallization of Polymer Semiconductors for Low-Temperature Fabrication
of High-Performance Organic Field-Effect Transistors. Adv. Funct. Mater..

[50] Snoek, J. ; Larochelle, H. ; Adams, R. P. Practical Bayesian Optimization of Machine Learning Algorithms. In NIPS’12: Proceedings of the 26th International Conference on Neural Information Processing Systems; Curran Associates, Inc.: Red Hook, NY, USA, 2012; pp 2951–2959.

[51] Lee, M. Classification_modeling_DPPDTT_OFETs; 2026. https://github.com/hahaha144/Classification_modeling_DPPDTT_OFETs. accessed 3 February 2026.

[52] Holliday S., Donaghey J. E., McCulloch I. (2014). Advances in Charge Carrier Mobilities
of Semiconducting Polymers Used in Organic Transistors. Chem. Mater..

[53] Kim H. S., Jeon S. H., Park J. S., Kim T. S., Son K. S., Seon J.-B., Seo S.-J., Kim S.-J., Lee E., Chung J. G., Lee H., Han S., Ryu M., Lee S. Y., Kim K. (2013). Anion Control as a Strategy to Achieve
High-Mobility and High-Stability Oxide Thin-Film Transistors. Sci. Rep..

[54] Bradley A. P. (1997). The Use
of the Area under the ROC Curve in the Evaluation of Machine Learning
Algorithms. Pattern Recognit..

[55] Brier G. W. (1950). Verification
of Forecasts Expressed in Terms of Probability. Mon. Weather Rev..

[56] Lee, B. SAXSLee; 2025. https://github.com/byeongdu/SAXSLee. accessed 3 February 2026.

[57] Ilavsky J. (2012). Nika: Software
for Two-Dimensional Data Reduction. J. Appl.
Crystallogr..

[58] Jiang Z. (2015). GIXSGUI: A
MATLAB Toolbox for Grazing-Incidence X-ray Scattering Data Visualization
and Reduction, and Indexing of Buried Three-Dimensional Periodic Nanostructured
Films. J. Appl. Crystallogr..

[59] Shimizu T., Whitfield R., Jones G. R., Raji I. O., Konkolewicz D., Truong N. P., Anastasaki A. (2023). Controlling Primary Chain Dispersity
in Network Polymers: Elucidating the Effect of Dispersity on Degradation. Chem. Sci..

[60] Wu Z., Yan Y., Zhao Y., Liu Y. (2022). Recent Advances in Realizing Highly
Aligned Organic Semiconductors by Solution-Processing Approaches. Small Methods.

[61] Dacha P., Hambsch M., Pohl D., Haase K., Löffler M., Lan T., Feng X., Rellinghaus B., Mannsfeld S. C. B. (2024). Tailoring
the Morphology of a Diketopyrrolopyrrole-based Polymer as Films or
Wires for High-Performance OFETs using Solution Shearing. Small Methods.

[62] Venkatesh R., Liu A. L., Zheng Y., Zhao H., Grover M. A., Meredith J. C., Reichmanis E. (2024). Harnessing
Compositional Gradients
to Elucidate Phase Behaviors toward High Performance Polymer Semiconductor
Blends. ACS Appl. Electron. Mater..

[63] Sharma A., Mathijssen S. G. J., Kemerink M., de Leeuw D. M., Bobbert P. A. (2009). Proton
Migration Mechanism for the Instability of Organic Field-Effect Transistors. Appl. Phys. Lett..

[64] Sowade E., Ramon E., Mitra K. Y., Martínez-Domingo C., Pedró M., Pallarès J., Loffredo F., Villani F., Gomes H. L., Terés L., Baumann R. R. (2016). All-Inkjet-Printed
Thin-Film Transistors: Manufacturing Process Reliability by Root Cause
Analysis. Sci. Rep..

[65] Hestand N. J., Spano F. C. (2018). Expanded Theory of H- and J-Molecular Aggregates: The
Effects of Vibronic Coupling and Intermolecular Charge Transfer. Chem. Rev..

[66] Spano F. C., Silva C. (2014). H- and J-Aggregate
Behavior in Polymeric Semiconductors. Annu.
Rev. Phys. Chem..

[67] Park K. S., Kwok J. J., Kafle P., Diao Y. (2021). When Assembly Meets
Processing: Tuning Multiscale Morphology of Printed Conjugated Polymers
for Controlled Charge Transport. Chem. Mater..

[68] Xu Z., Saiev S., Qian P., Nabei Y., Wang Z., Rinehart J. M., Österholm A. M., Jones A. L., Lee J.-H., Hwang C., Wang S., Sun R., Shin D., Jeon S., Elangovan K. E., Vura-Weis J., Coropceanu V., Rodríguez-López J., Reynolds J. R., Sun D., Brédas J.-L., Diao Y. (2025). Supramolecular Chirality Largely
Modulates Chemical Doping of Conjugated
Polymers. Nat. Commun..

[69] Deng J., Zheng L., Ding C., Guo Y., Xie Y., Wang J., Ke Y., Li M., Li L., Janssen R. A. J. (2023). Determinant Role of Solution-State Supramolecular Assembly
in Molecular Orientation of Conjugated Polymer Films. Adv. Funct. Mater..

[70] Noriega R., Rivnay J., Vandewal K., Koch F. P., Stingelin N., Smith P., Toney M. F., Salleo A. (2013). A General Relationship
between Disorder, Aggregation and Charge Transport in Conjugated Polymers. Nat. Mater..

[71] Zheng Y., Venkatesh R., Callaway C. P., Viersen C., Fagbohungbe K. H., Liu A. L., Risko C., Reichmanis E., Silva-Acuña C. (2023). Chain Conformation and Exciton Delocalization in a
Push-Pull Conjugated Polymer. Chem. Mater..

[72] Xu Z., Park K. S., Kwok J. J., Lin O., Patel B. B., Kafle P., Davies D. W., Chen Q., Diao Y. (2022). Not All Aggregates
Are Made the Same: Distinct Structures of Solution Aggregates Drastically
Modulate Assembly Pathways, Morphology, and Electronic Properties
of Conjugated Polymers. Adv. Mater..

[73] Park M. S., Aiyar A., Park J. O., Reichmanis E., Srinivasarao M. (2011). Solvent Evaporation Induced Liquid
Crystalline Phase
in poly­(3-hexylthiophene). J. Am. Chem. Soc..

[74] Kleinhenz N., Rosu C., Chatterjee S., Chang M., Nayani K., Xue Z., Kim E., Middlebrooks J., Russo P. S., Park J. O., Srinivasarao M., Reichmanis E. (2015). Liquid Crystalline Poly­(3-hexylthiophene)
Solutions Revisited: Role of Time-Dependent Self-Assembly. Chem. Mater..

