# Supplementary material for: Data-Driven Discovery and Experimental Validation of Solvent Polarity Effects on Conjugated Polymer Solution-to-Film Assembly Pathways
Source: Chem Mater. 2026 Jul 16;38(14):7130–40. doi: 10.1021/acs.chemmater.6c00358 (PMC13420547; doi:10.1021/acs.chemmater.6c00358)
Supplement: Supplementary file 1 [file cm6c00358_si_001.pdf]

Supporting Information

Data-Driven Discovery and Experimental Validation of Solvent Polarity Effects on Conjugated Polymer Solution-to-Film Assembly Pathways

Myeongyeon Lee,[a]† *Sanghyun Jeon,*[b]† *Yen-Chi Chen,*[c] *Priyotosh Bairagya,*[c] *Chiwon Yu,*[a] *Azzaya Khasbaatar,*[c] *Nahyun Ahn,*[c] *Ying Diao,*[c,*] *Martha A. Grover,*[d,*] *Elsa Reichmanis*[a,*]

[a]Department of Chemical and Biomolecular Engineering, Lehigh University, Bethlehem, Pennsylvania 18015, United States

[b]Department of Materials Science and Engineering, University of Illinois at Urbana-Champaign, 1304 West Green Street, Urbana, Illinois 61801, United States

[c]Department of Chemical and Biomolecular Engineering, University of Illinois at Urbana-Champaign, 600 South Mathews Avenue, Urbana, Illinois 61801, United States

[d]School of Chemical & Biomolecular Engineering, Georgia Institute of Technology, 311 Ferst Drive, Atlanta, Georgia 30332, United States

† These authors contributed equally to this work.

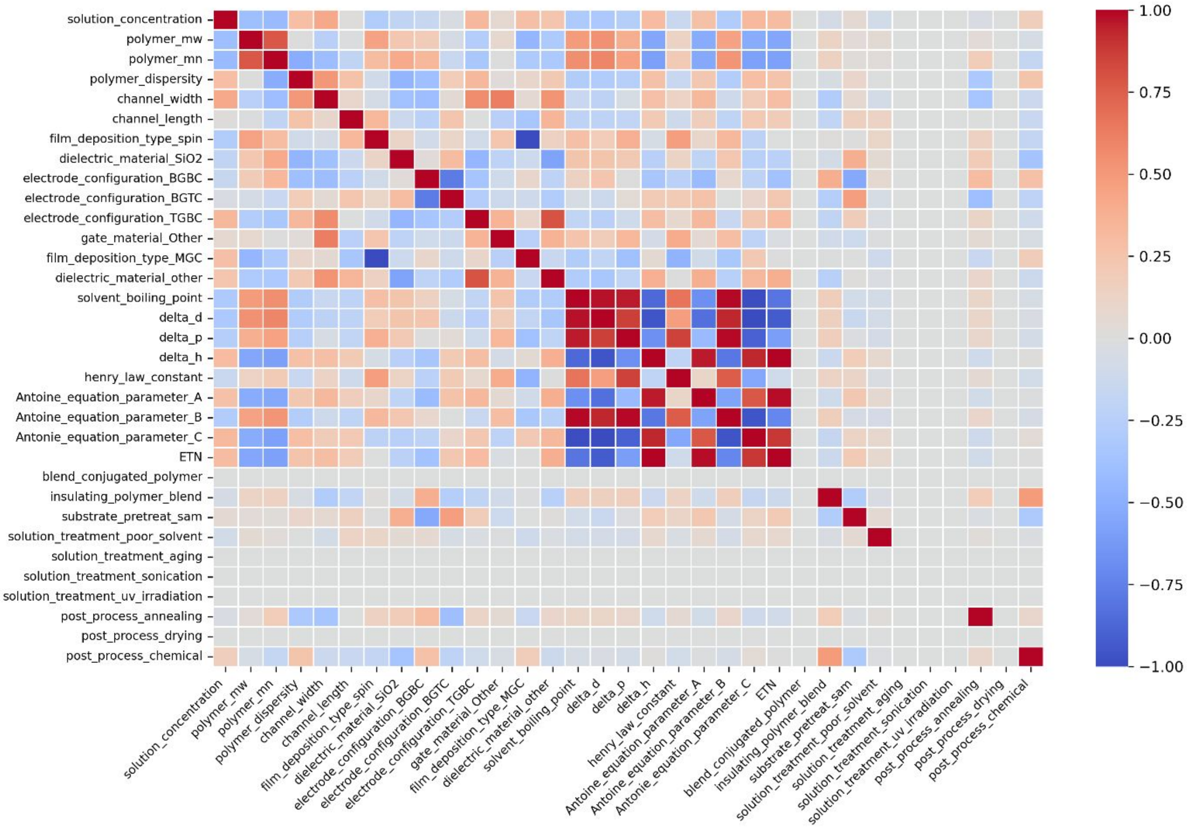

Figure S1. Pearson correlation matrix of the extracted parameters from the database.

Table S1. Removed process parameters and removal reason

Process parameter	Reason for Removal	Support
Blend conjugated polymer	All values are 0	
Solution treatment aging	All values are 0	
Solution treatment sonication	All values are 0	
Solution treatment UV irradiation	All values are 0	
Post process drying	All values are 0	
Polymer number average molecular weight (M_n)	Dependence of PDI and Mw	PDI = Mw/Mn
Channel width	The value is already included in the charge mobility equation	$I_{DS}=(W\cdot C\cdot \mu/2\cdot L)(V_G-V_T)^2$
Channel length	The value is already included in the charge mobility equation	$I_{DS}=(W\cdot C\cdot \mu/2\cdot L)(V_G-V_T)^2$
Spin coating	The value is correlated with meniscus-guided coating	
Dielectric material SiO_2	The value is already included in the charge mobility equation	$I_{DS}=(W\cdot C\cdot \mu/2\cdot L)(V_G-V_T)^2$
Dielectric material other	The value is already included in the charge mobility equation	$I_{DS}=(W\cdot C\cdot \mu/2\cdot L)(V_G-V_T)^2$
Electrode configuration BGBC	structural parameters unrelated to the conjugated polymer film	
Electrode configuration BGTC	structural parameters unrelated to the conjugated polymer film	
Electrode configuration TGBC	structural parameters unrelated to the conjugated polymer film	
Gate material other	structural parameters unrelated to the conjugated polymer film	
Hansen solubility delta p	The value is correlated with other solvent parameters	
Hansen solubility delta d	The value is correlated with other solvent parameters	
Hansen solubility delta h	The value is correlated with other solvent parameters	
Henry's law constant	The value is correlated with other solvent parameters	
Anotoine equation parameter A	The value is correlated with other solvent parameters	
Anotoine equation parameter B	The value is correlated with other solvent parameters	
Anotoine equation parameter C	The value is correlated with other solvent parameters	
Solvent boiling point	The value is correlated with other solvent parameters	

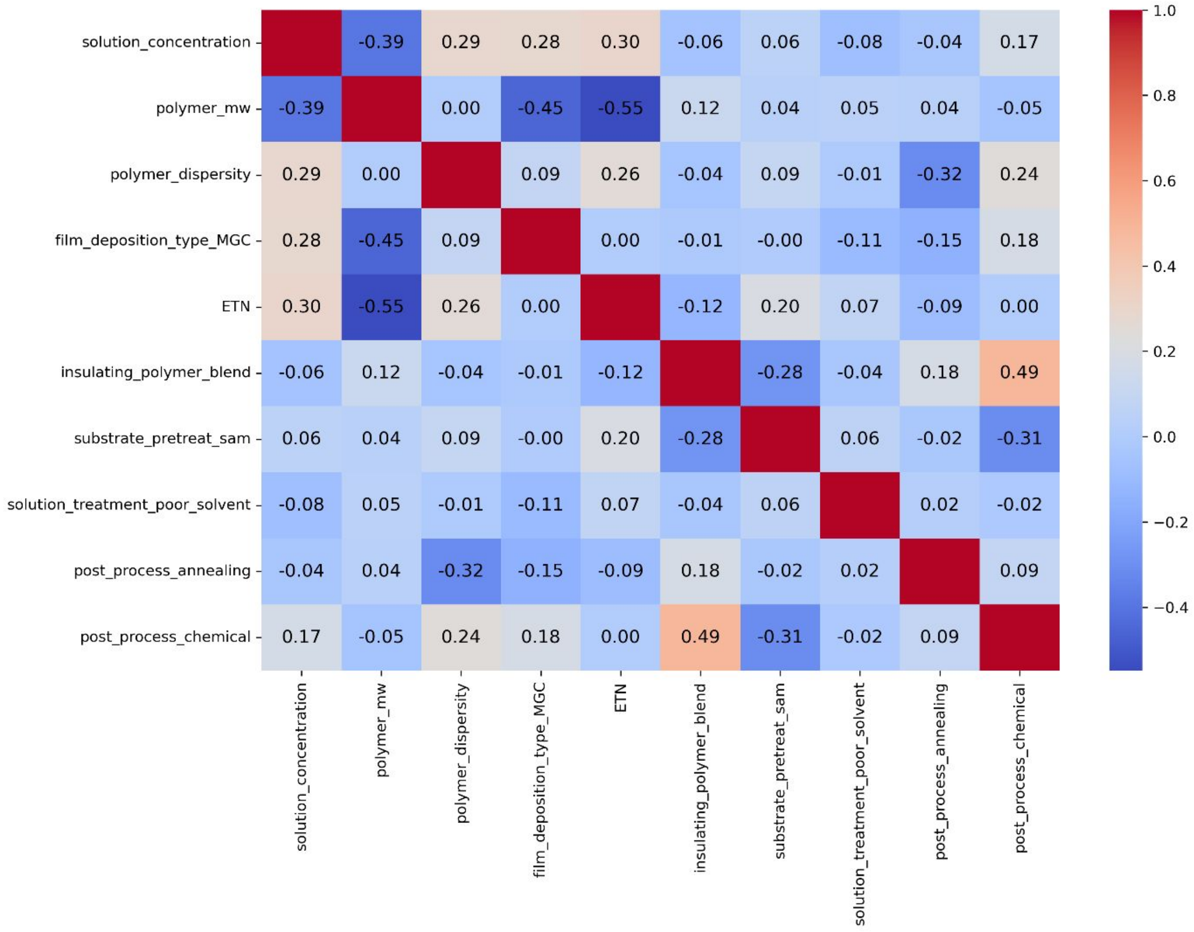

Figure S2. Pearson correlation matrix of the selected parameters

Table S2. Class distribution of the dataset at different mobility thresholds

Threshold mobility (cm^2/Vs)	Total samples	Total low	Total high	Train samples	Train low	Train high	Test samples	Test low	Test high
0.2	204	119	85	163	95	68	41	24	17
0.4		154	50		123	40		31	10
0.6		163	41		130	33		33	8
0.8		174	30		139	24		35	6
1.0		174	30		139	24		35	6
1.2		176	28		141	22		35	6
1.4		181	23		145	18		35	6
1.6		182	22		145	18		37	4
1.8		182	22		145	18		37	4
2.0		184	20		147	16		37	4

Table S3. Model setup, hyperparameter search space, and optimized values

Classifier	Hyperparameters	Search Space	Optimized Value	Fixed Parameters
Logistics	max_iter	Integer (10, 5000)	2056	class_weight="balanced" solver="lbfgs" penalty="l2" random_state=42
Ridge	alpha	Real (0.1, 1.0, prior="log-uniform")	0.2571	class_weight="balanced" random_state=42
	max_iter	Integer(10, 5000)	3641	
SGD	alpha	Real (0.1, 1.0, prior="log-uniform")	0.1181	loss="log_loss" class_weight="balanced" random_state=42
	max_iter	Integer (10, 5000)	5000	
Decision Tree	max_depth	Integer (2, 50)	50	class_weight="balanced" random_state=42
	min_samples_leaf	Integer (1, 50)	1	
	min_samples_split	Integer (2, 100)	2	
Random Forest	n_estimators	Integer (10, 5000)	548	class_weight="balanced" random_state=42 n_jobs=-1
	max_depth	Integer (2, 50)	50	
	min_samples_split	Integer (2, 100)	2	
	min_samples_leaf	Integer (1, 20)	1	
	max_features	Real (0.2, 1.0)	0.9514	
Extra Tree	n_estimators	Integer (10, 5000)	5000	class_weight="balanced" random_state=42 n_jobs=-1
	max_depth	Integer (2, 50)	50	
	min_samples_split	Integer (2, 100)	7	
	min_samples_leaf	Integer (1, 20)	3	
	max_features	Real (0.2, 1.0)	0.2	

Gradient Boost	n_estimators	Integer (10, 5000)	4992	loss="log_loss" random_state=42
	learning_rate	Rea l(1e-3, 1.0, prior="log-uniform")	0.0068	
	max_depth	Integer(2, 8)	4	
	subsample	Real (0.5, 1.0)	0.7216	
	min_sample_leaf	Integer (1, 50)	11	
	max_features	Real(0.2, 1.0)	1	
Hist Gradient Boost	max_iter	Integer(100, 5000)	100	loss="log_loss" random_state=42
	learning_rate	Real (1e-3, 1.0, prior="log-uniform")	0.2350	
	max_depth	Integer (2, 16)	2	
	max_leaf_nodes	Integer (2, 255)	255	
	min_samples_leaf	Integer (1, 100)	8	
	l2_regularization	Real (1e-8, 1.0, prior="log-uniform")	1.0	
	max_bins	Integer(2, 255)	255	
XGBoost	n_estimators	Integer (10, 5000)	10	objective="binary:logstics" eval_metric="auc" tree_method="hist" n_jobs=-1 random_state=42
	learning_rate	Real (1e-3, 1.0, prior="log-uniform")	1.0	
	max_depth	Integer (2, 16)	16	
	min_child_weight	Integer (1, 20)	1	
	subsample	Real (0.1, 1.0)	0.6687	

	colsample_bytree	Real (0.2, 1.0)	0.2	
	gamma	Rea l(0.0, 1.0)	0.0	
	reg_alpha	Real (1e-8, 1.0, prior="log-uniform")	1e-8	
	reg_lamda	Real (1e-8, 1.0, prior="log-uniform")	1e-8	
LightGBM	n_estimators	Integer (10, 5000)	10	Objective="binary" Boosting_type="gbdt" is_unbalance=True n_jobs=-1 random_state=42
	learning_rate	Real (1e-3, 1.0, prior="log-uniform")	1.0	
	max_depth	Integer (2, 16)	16	
	min_child_samples	Integer (1, 200)	1	
	min_child_weight	Real (1e-3, 100.0, prior="log-uniform")	0.0018	
	subsample	Real (0.1, 1.0)	1.0	
	colsample_bytree	Real (0.1, 1.0)	0.1	
	max_bin	Integer (2, 512)	16	
CatBoost	interations	Integer (10, 5000)	4773	
	learning_rate	Real (1e-3, 1.0, prior="log-uniform")	1.0	
	depth	Integer (2, 10)	2	
	l2_leaf_reg	Real (1e-3, 100.0, prior="log-uniform")	0.001	
	min_data_in_leaf	Integer (1, 200)	5	

	rsm	Real (0.2, 1.0)	0.3922	
	subsample	Real (0.5, 1.0)	0.5	
AdaBoost	n_estimators	Integer (1, 5000)	1241	estimator=base_tree algorithm="SAMME.R" random_state=42
	learning_rate	Real (1e-3, 1.0, prior="log-uniform")	0.011	
	estimator_max_depth	Integer (1, 10)	5	
	estimator_min_samples_leaf	Integer (1, 50)	31	
	estimator_min_samples_split	Integer (2, 100)	76	
	estimator_max_features	Real (0.2, 1.0)	0.6474	
MLP	activation	Categorical (["relu", "tanh", "logstic"])	Relu	hidden_layer_size=(256,) Solver="adam" early_stopping=True validation_fraction=0.1 n_iter_no_change=5 random_state=42
	alpha	Real (1e-6, 1e-1, prior="log-uniform")	1e-6	
	learning_rate_init	Real (1e-4, 1e-1, prior="log-uniform")	0.0444	
	batch_size	Categorical (["32", "64", "128", "256"])	64	
	max_iter	Integer (10,5000)	10	
SVM	svc_C	Real (1e-3, 1e3, prior="log-uniform")	212.594	kernal="rbf" class_weight="balanced" probability=Ture random_state=42
	svc_gamma	Real (1e-4, 1e1, prior="log-uniform")	0.00018	

svc_shrinking	Categorical ([True,False])	True	
svc_max_iter	Integer (100,5000)	4778	

Table S4. Bayesian optimization and cross-validation settings

Parameter	Value/Description
cross-validation	Stratified K-fold (n_split=5, shuffle=True, radom_state=42)
iterations	50 (logistic, ridge, SGD, Decision Tree); 60 (Gradient Boost, MLP); 100 (Random Forest, Extra Tree, SVM); 200 (Hist Gradient Boost, XGBoost, LightGBM, CatBoost, AdaBoost)
refit	True
score	ROC-AUC
random_state	42
n_jobs	-1
return_train_score	False

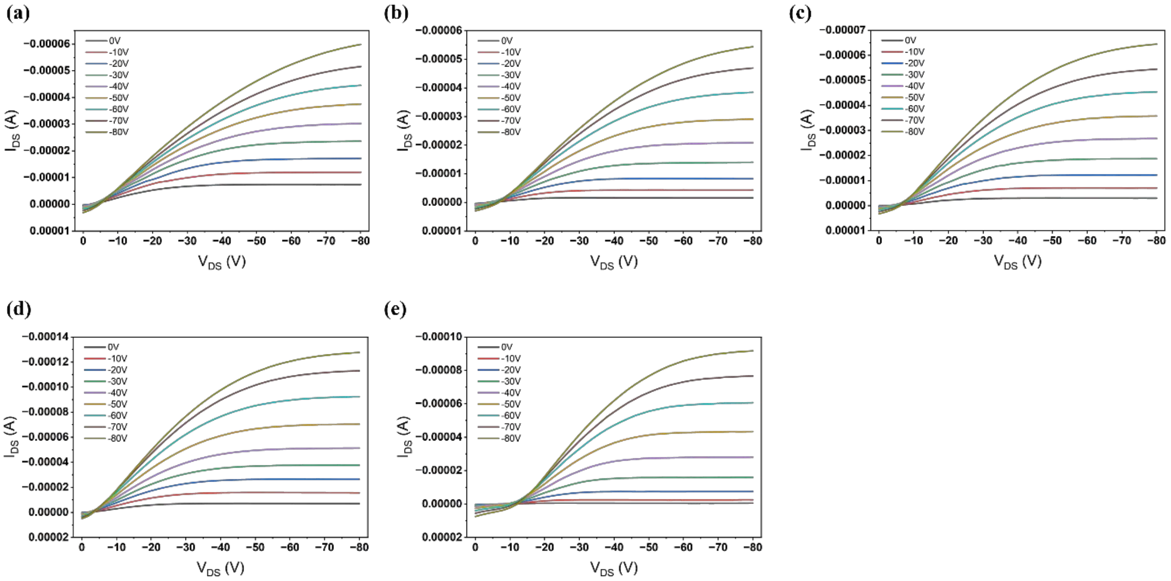

Figure S3. Output curve of (a) XY, (b) TOL, (c) DCB, (d) CB, and (e) CF.

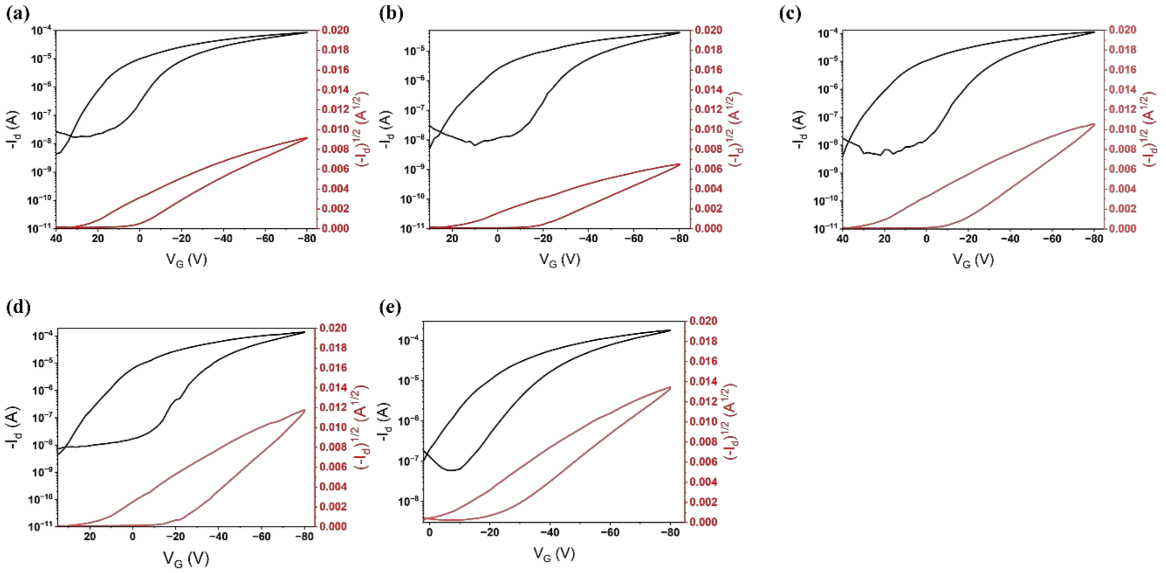

Figure S4. Transfer curve of (a) XY, (b) TOL, (c) DCB, (d) CB, and (e) CF.

Table S5. Threshold sensitivity analysis of model ROC-AUC

Threshold mobility (cm^2/Vs)	0.2	0.4	0.6	0.8	1.0	1.2	1.4	1.6	1.8	2.0
Model										
AdaBoost	0.9379 ±0.0433	0.9064 ±0.0884	0.9780 ±0.0205	0.9932 ±0.0094	0.9932 ±0.0094	0.9717 ±0.0259	1.0000 ±0.0000	1.0000 ±0.0000	1.0000 ±0.0000	0.9832 ±0.0190
CatBoost	0.9034 ±0.0445	**0.9711 ±0.0228**	0.9966 ±0.0058	0.9977 ±0.0038	0.9977 ±0.0038	0.9717 ±0.0259	1.0000 ±0.0000	**1.0000 ±0.0000**	**1.0000 ±0.0000**	0.9762 ±0.0246
Decision Tree	0.8836 ±0.0524	0.9164 ±0.0445	0.9365 ±0.0423	0.9104 ±0.0883	0.9100 ±0.0905	0.9783 ±0.0201	1.0000 ±0.0000	**1.0000 ±0.0000**	**1.0000 ±0.0000**	0.9565 ±0.0309
Extra Trees	0.9272 ±0.0466	0.9711 ±0.0228	0.9896 ±0.0125	0.9977 ±0.0038	0.9977 ±0.0038	**0.9788 ±0.0196**	0.9775 ±0.0234	1.0000 ±0.0000	1.0000 ±0.0000	0.9762 ±0.0246
Gradient Boosting	0.9451 ±0.0419	0.9146 ±0.0553	0.9780 ±0.0205	**0.9977 ±0.0038**	**0.9977 ±0.0038**	0.9717 ±0.0259	**1.0000 ±0.0000**	**1.0000 ±0.0000**	**1.0000 ±0.0000**	0.9762 ±0.0246
Hist Gradient Boosting	**0.9457 ±0.0328**	0.9052 ±0.0636	0.9780 ±0.0205	0.9932 ±0.0094	0.9932 ±0.0094	0.9717 ±0.0259	1.0000 ±0.0000	**1.0000 ±0.0000**	**1.0000 ±0.0000**	0.9762 ±0.0246
LightGBM	0.9162 ±0.0475	0.9404 ±0.0547	0.9772 ±0.0221	0.9932 ±0.0094	0.9932 ±0.0094	0.9162 ±0.0475	1.0000 ±0.0000	1.0000 ±0.0000	1.0000 ±0.0000	0.9762 ±0.0246
Logistic	0.8777 ±0.0563	0.8969 ±0.0506	0.9586 ±0.0317	0.9926 ±0.0099	0.9926 ±0.0099	0.9717 ±0.0259	1.0000 ±0.0000	1.0000 ±0.0000	1.0000 ±0.0000	0.9768 ±0.0235
MLP	0.9026 ±0.0502	0.9302 ±0.0393	**1.0000 ±0.0000**	0.9802 ±0.0189	0.9802 ±0.0189	0.9585 ±0.0305	0.9889 ±0.0152	0.9929 ±0.0126	0.9929 ±0.0126	0.9762 ±0.0246
Random Forest	0.9175 ±0.0501	0.9428 ±0.0351	0.9780 ±0.0205	0.9935 ±0.0088	0.9935 ±0.0088	0.9764 ±0.0217	0.9941 ±0.0102	1.0000 ±0.0000	1.0000 ±0.0000	0.9697 ±0.0273
Ridge	0.8779 ±0.0548	0.8978 ±0.0493	0.9635 ±0.0276	0.9935 ±0.0088	0.9935 ±0.0088	0.9717 ±0.0259	0.9881 ±0.0163	1.0000 ±0.0000	1.0000 ±0.0000	0.9696 ±0.0272
SGD	0.9043 ±0.0507	0.9143 ±0.0468	0.9555 ±0.0333	0.9935 ±0.0088	0.9935 ±0.0088	0.9717 ±0.0259	0.9830 ±0.0200	0.9929 ±0.0126	0.9929 ±0.0126	0.9629 ±0.0292
SVM	0.8888 ±0.0609	0.9578 ±0.0324	0.9927 ±0.0098	0.9935 ±0.0088	0.9935 ±0.0088	0.9717 ±0.0259	0.9889 ±0.0152	0.9791 ±0.0228	0.9791 ±0.0228	**0.9895 ±0.0140**
XGBoost	0.8931 ±0.0546	0.9492 ±0.0380	0.9897 ±0.0130	0.9932 ±0.0094	0.9932 ±0.0094	0.9671 ±0.0272	0.9889 ±0.0152	1.0000 ±0.0000	1.0000 ±0.0000	0.9762 ±0.0246

*Values are reported as test set bootstrap average ± standard deviation. Bold values indicate the most accurate model based on ROC-AUC and Brier score

Table S6. Threshold sensitivity analysis of model Brier score

Threshold mobility (cm^2/Vs) Model	0.2	0.4	0.6	0.8	1.0	1.2	1.4	1.6	1.8	2.0
AdaBoost	0.2402	0.1741	0.1696	0.0398	0.0398	0.0410	0.0362	0.0230	0.0230	0.0401
CatBoost	0.2111	**0.2430**	0.0297	0.0177	0.0177	0.0387	0.0232	**0.0157**	**0.0157**	0.0400
Decision Tree	0.1328	0.1157	0.0797	0.0395	0.0395	0.0389	0.0237	**0.0157**	**0.0157**	0.0799
Extra Trees	0.1094	0.0594	0.0254	0.0265	0.0265	**0.0399**	0.0987	0.0158	0.0158	0.0449
Gradient Boosting	0.0771	0.0950	0.0421	**0.0168**	**0.0168**	0.0391	**0.0205**	**0.0157**	**0.0157**	0.0524
Hist Gradient Boosting	**0.1008**	0.1131	0.0461	0.0316	0.0316	0.0389	0.0237	**0.0157**	**0.0157**	0.0557
LightGBM	0.1041	0.0867	0.0390	0.0383	0.0383	0.0393	0.0229	0.0159	0.0159	0.0318
Logistic	0.1363	0.1298	0.0788	0.0289	0.0289	0.0542	0.0287	0.0209	0.0209	0.0444
MLP	0.1484	0.1164	**0.1252**	0.0453	0.0453	0.0533	0.1309	0.0976	0.0976	0.0246
Random Forest	0.1103	0.0935	0.0589	0.0202	0.0202	0.0388	0.0264	0.0159	0.0159	0.0639
Ridge	0.1485	0.1432	0.1015	0.1400	0.1400	0.1626	0.1229	0.1296	0.1296	0.1729
SGD	0.1927	0.1374	0.1021	0.0577	0.0577	0.0753	0.0474	0.0685	0.0685	0.1229
SVM	0.1240	0.0668	0.0490	0.0378	0.0378	0.0586	0.0335	0.0220	0.0220	**0.0355**
XGBoost	0.1353	0.0844	0.0199	0.0316	0.0316	0.0432	0.2467	0.0169	0.0169	0.0576

*Values are reported as test set Brier score. Bold values indicate the most accurate model based on ROC-AUC and Brier score

Table S7. Performance metrics including average ROC-AUC, standard deviation, and Brier score for each ML classifier under grouped samples

Classifier Model	Average ROC-AUC	Standard Deviation	Brier Score
**Decision Tree**	**1.0000**	**0.0000**	**0.0585**
SGD	1.0000	0.0000	0.0630
Random Forest	1.0000	0.0000	0.0698
SVM	1.0000	0.0000	0.0942
MLP	1.0000	0.0000	0.0947
XGBoost	1.0000	0.0000	0.1136
Extra Trees	1.0000	0.0000	0.1147
Hist Gradient Boosting	1.0000	0.0000	0.1440
Logistic	0.8648	0.0448	0.1282
Gradient Boosting	0.8403	0.1049	0.1455
LightGBM	0.8144	0.0934	0.1401
AdaBoost	0.8082	0.0576	0.1421
Ridge	0.7253	0.0735	0.5689
CatBoost	0.3990	0.0730	0.1438

Table S8. Model setup, hyperparameter search space, and optimized values under grouped samples

Classifier	Hyperparameters	Search Space	Optimized Value	Fixed Parameters
Logistics	max_iter	Integer (10, 5000)	2056	class_weight="balanced" solver="lbfgs" penalty="l2" random_state=42
Ridge	alpha	Real (0.1, 1.0, prior="log-uniform")	0.6877	class_weight="balanced" random_state=42
	max_iter	Integer(10, 5000)	4418	
SGD	alpha	Real (0.1, 1.0, prior="log-uniform")	0.1262	loss="log_loss" class_weight="balanced" random_state=42
	max_iter	Integer (10, 5000)	4851	
Decision Tree	max_depth	Integer (2, 50)	49	class_weight="balanced" random_state=42
	min_samples_leaf	Integer (1, 50)	12	
	min_samples_split	Integer (2, 100)	2	
Random Forest	n_estimators	Integer (10, 5000)	548	class_weight="balanced" random_state=42 n_jobs=-1
	max_depth	Integer (2, 50)	20	
	min_samples_split	Integer (2, 100)	2	
	min_samples_leaf	Integer (1, 20)	5	
	max_features	Real (0.2, 1.0)	0.4823	
Extra Tree	n_estimators	Integer (10, 5000)	5000	class_weight="balanced" random_state=42 n_jobs=-1
	max_depth	Integer (2, 50)	50	
	min_samples_split	Integer (2, 100)	2	
	min_samples_leaf	Integer (1, 20)	4	
	max_features	Real (0.2, 1.0)	0.2	

Gradient Boost	n_estimators	Integer (10, 5000)	4304	loss="log_loss" random_state=42
	learning_rate	Rea l(1e-3, 1.0, prior="log-uniform")	0.0083	
	max_depth	Integer(2, 8)	8	
	subsample	Real (0.5, 1.0)	0.8766	
	min_sample_leaf	Integer (1, 50)	1	
	max_features	Real(0.2, 1.0)	1	
Hist Gradient Boost	max_iter	Integer(100, 5000)	527	loss="log_loss" random_state=42
	learning_rate	Real (1e-3, 1.0, prior="log-uniform")	1.0	
	max_depth	Integer (2, 16)	12	
	max_leaf_nodes	Integer (2, 255)	165	
	min_samples_leaf	Integer (1, 100)	28	
	l2_regularization	Real (1e-8, 1.0, prior="log-uniform")	1e-08	
	max_bins	Integer(2, 255)	5	
XGBoost	n_estimators	Integer (10, 5000)	5000	objective="binary:logstics" eval_metric="auc" tree_method="hist" n_jobs=-1 random_state=42
	learning_rate	Real (1e-3, 1.0, prior="log-uniform")	0.001	
	max_depth	Integer (2, 16)	2	
	min_child_weight	Integer (1, 20)	10	
	subsample	Real (0.1, 1.0)	0.8108	

	colsample_bytree	Real (0.2, 1.0)	0.2	
	gamma	Rea l(0.0, 1.0)	10.0	
	reg_alpha	Real (1e-8, 1.0, prior="log-uniform")	0.0003	
	reg_lamda	Real (1e-8, 1.0, prior="log-uniform")	1e-8	
LightGBM	n_estimators	Integer (10, 5000)	1896	Objective="binary" Boosting_type="gbdt" is_unbalance=True n_jobs=-1 random_state=42
	learning_rate	Real (1e-3, 1.0, prior="log-uniform")	0.2285	
	max_depth	Integer (2, 16)	5	
	min_child_samples	Integer (1, 200)	1	
	min_child_weight	Real (1e-3, 100.0, prior="log-uniform")	0.9097	
	subsample	Real (0.1, 1.0)	0.3754	
	colsample_bytree	Real (0.1, 1.0)	0.4008	
	max_bin	Integer (2, 512)	2	
CatBoost	interations	Integer (10, 5000)	807	
	learning_rate	Real (1e-3, 1.0, prior="log-uniform")	1.0	
	depth	Integer (2, 10)	10	
	l2_leaf_reg	Real (1e-3, 100.0, prior="log-uniform")	0.0359	
	min_data_in_leaf	Integer (1, 200)	132	

	rsm	Real (0.2, 1.0)	1.0	
	subsample	Real (0.5, 1.0)	0.5	
AdaBoost	n_estimators	Integer (1, 5000)	2965	estimator=base_tree algorithm="SAMME.R" random_state=42
	learning_rate	Real (1e-3, 1.0, prior="log-uniform")	0.008	
	estimator_max_depth	Integer (1, 10)	8	
	estimator_min_samples_leaf	Integer (1, 50)	29	
	estimator_min_samples_split	Integer (2, 100)	2	
	estimator_max_features	Real (0.2, 1.0)	0.5012	
MLP	activation	Categorical (["relu", "tanh", "logstic"])	Logistic	hidden_layer_size=(256,) Solver="adam" early_stopping=True validation_fraction=0.1 n_iter_no_change=5 random_state=42
	alpha	Real (1e-6, 1e-1, prior="log-uniform")	0.0568	
	learning_rate_init	Real (1e-4, 1e-1, prior="log-uniform")	0.0002	
	batch_size	Categorical (["32", "64", "128", "256"])	64	
	max_iter	Integer (10,5000)	5000	
SVM	svc_C	Real (1e-3, 1e3, prior="log-uniform")	0.4081	kernal="rbf" class_weight="balanced" probability=Ture random_state=42
	svc_gamma	Real (1e-4, 1e1, prior="log-uniform")	0.0556	

svc_shrinking	Categorical ([True,False])	True
svc_max_iter	Integer (100,5000)	632

Table S9. Bayesian optimization and grouped-aware cross-validation settings

Parameter	Value/Description
cross-validation	Group-aware K-fold (n_split=5, shuffle=True, radom_state=42)
iterations	50 (logistic, ridge, SGD, Decision Tree); 60 (Gradient Boost, MLP); 100 (Random Forest, Extra Tree, SVM); 200 (Hist Gradient Boost, XGBoost, LightGBM, CatBoost, AdaBoost)
refit	True
score	ROC-AUC
random_state	42
n_jobs	-1
return_train_score	False

Table S10. Relative importance of process parameters estimated by the Decision Tree classifier using permutation analysis with grouped samples

Process parameter	Permutation importance
Film deposition MGC	0.50953
Solution concentration	0.00000
Weight average molecular weight	0.00000
Polymerdispersity index	0.00000
E_T^N	0.00000
Insulating polymer blend	0.00000
Substrate pretreatment SAM	0.00000
Solution treatment poor solvent	0.00000
Annealing	0.00000
Post chemical process	0.00000

Table S11. Regression model performance analysis

Model	Average train set R^2	Standard deviation train set R^2	Average test set R^2	Standard deviation test set R^2
AdaBoost	0.5706	0.5403	0.6284	0.1958
CatBoost	0.5833	0.6529	0.6709	0.2252
Decision Tree	0.5442	0.7170	-1.7389	4.6332
Elastic net	0.2375	0.4685	-1.0130	2.2991
Extra Trees	0.5691	0.7252	0.6037	0.3123
Gradient Boost	0.5186	0.8084	0.2049	0.9601
Hist Gradient Boost	0.5137	0.7510	0.7418	0.1266
Lasso	0.1652	0.6198	-1.2218	2.6324
Light GBM	0.5434	0.7082	-0.1683	1.7937
MLP	0.5547	0.6777	0.4993	0.4636
Ordinary	0.1146	0.8954	-2.2570	4.0270
Random Forest	0.5821	0.6478	0.7764	0.1236
Ridge	0.2375	0.4749	-1.1587	2.5150
SGD	0.2473	0.4559	-0.9464	2.1946
SVM	0.5014	0.9064	0.5137	0.5520
XGBoost	0.5061	0.7426	-0.0921	1.5513

Table S12. Relative importance of process parameters estimated by the Gradient Boosting classifier using permutation analysis.

Process parameter	Permutation importance
Weight average molecular weight	0.04158730
Polymerdispersity index	0.03992063
Film deposition MGC	0.01746032
E_T^N	0.00928571
Solution concentration	0.00476191
Substrate pretreatment SAM	0.00126984
Insulating polymer blend	0.00126984
Annealing	0.00000000
Post chemical process	0.00000000
Solution treatment poor solvent	0.00000000

Table S13. DPP-DTT solubility test of various solvents at 5 mg/mL.

Solvent	Affinity
Acetone	Bad
Acetonitrile	Bad
Anisole	Bad
Chlorobenzene	Good
Chloroform	Good
1-Chloronaphthalene	Good
Decane	Bad
o-dichlorobenzene	Good
1,4-dichlorobutane	Bad
Dimethyl Sulfoxide	Bad
Ethanol	Bad
Ethyl acetate	Bad
Ethyl dichloride	Bad
Mesitylene	Bad
Methanol	Bad
Methylene dichloride	Bad
2-propanol	Bad
Tetrachloroethylene	Good
Tetrahydrofuran	Bad
Toluene	Good
1,2,4-Trichlorobenzene	Good
Trichloroethylene	Good
Water	Bad
p-xylene	Good

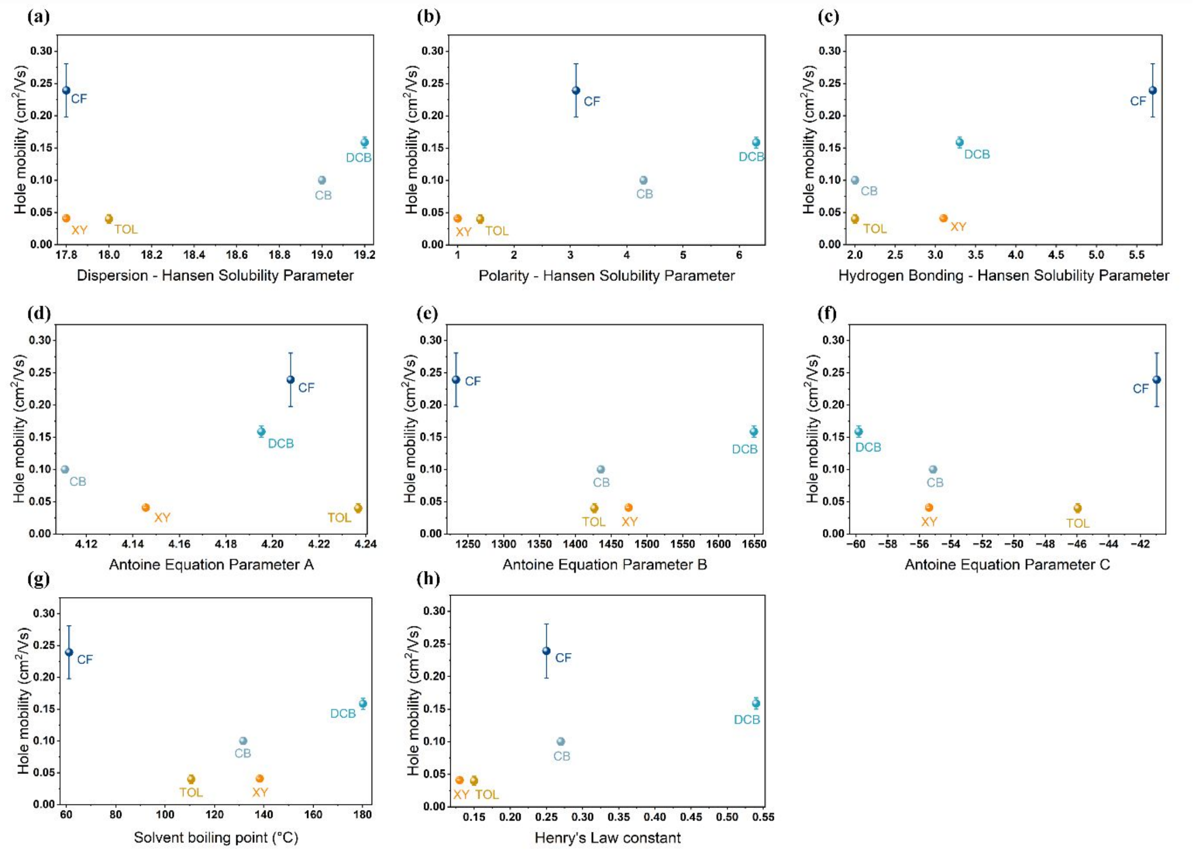

Figure S5. Hole mobility of DPP-DTT OFETs as a function of solvent parameters: Hansen solubility parameters (a) dispersion (δD), (b) polarity (δP), (c) hydrogen bonding (δH) (unit $MPa^{1/2}$), (d-f) Antoine equation parameters (A-C), (g) solvent boiling point, and (h) Henry's law constant. Error bars here represent the standard deviation obtained from 8-12 OFET devices.

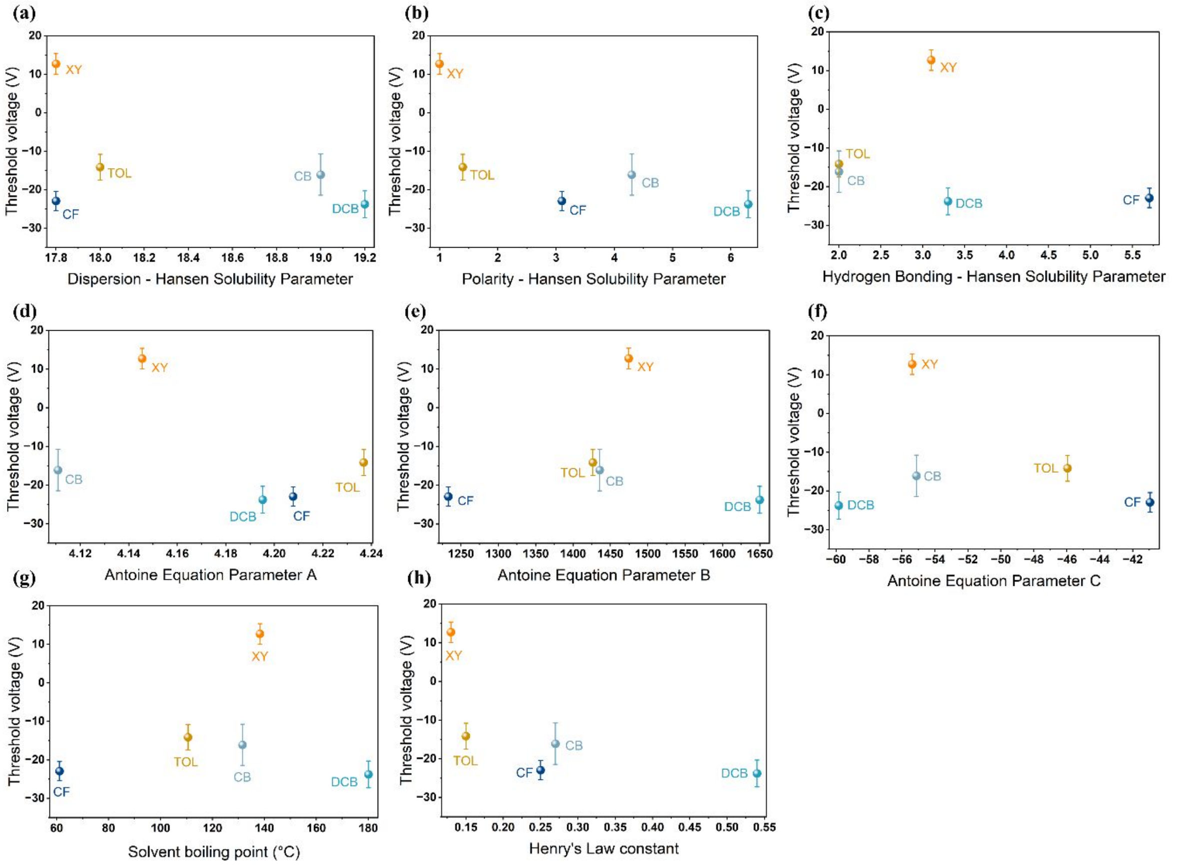

Figure S6. Threshold voltage of DPP-DTT OFETs as a function of solvent parameters: Hansen solubility parameters (a) dispersion (δD), (b) polarity (δP), (c) hydrogen bonding (δH) (unit $MPa^{1/2}$), (d-f) Antoine equation parameters (A-C), (g) solvent boiling point, and (h) Henry's law constant. Error bars here represent the standard deviation obtained from 8-12 OFET devices.

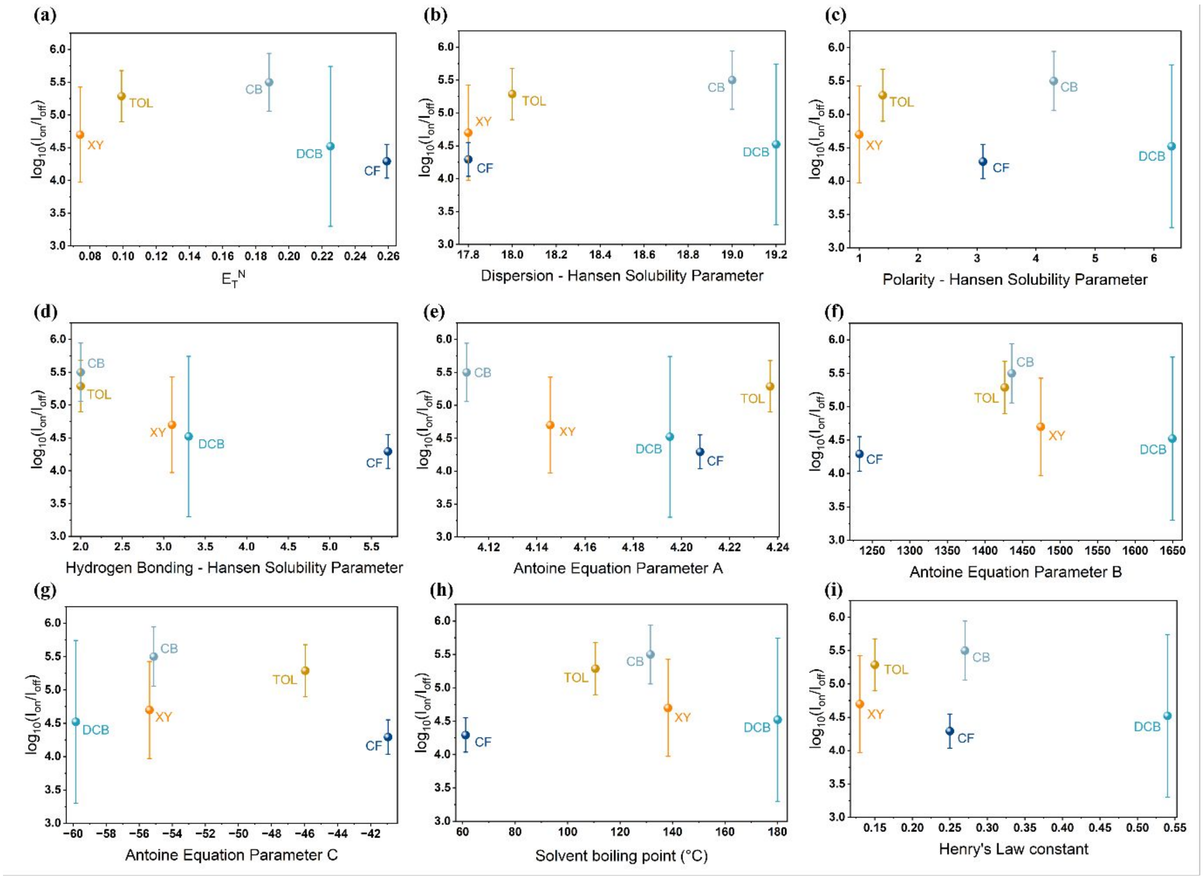

Figure S7. Current on/off ratio of DPP-DTT OFETs as a function of solvent parameters: (a) E_T^N, Hansen solubility parameters (b) dispersion (δD), (c) polarity (δP), (d) hydrogen bonding (δH) (unit $MPa^{1/2}$), (e-g) Antoine equation parameters (A-C), (h) solvent boiling point, and (i) Henry's law constant. Error bars here represent the standard deviation obtained from 8-12 OFET devices.

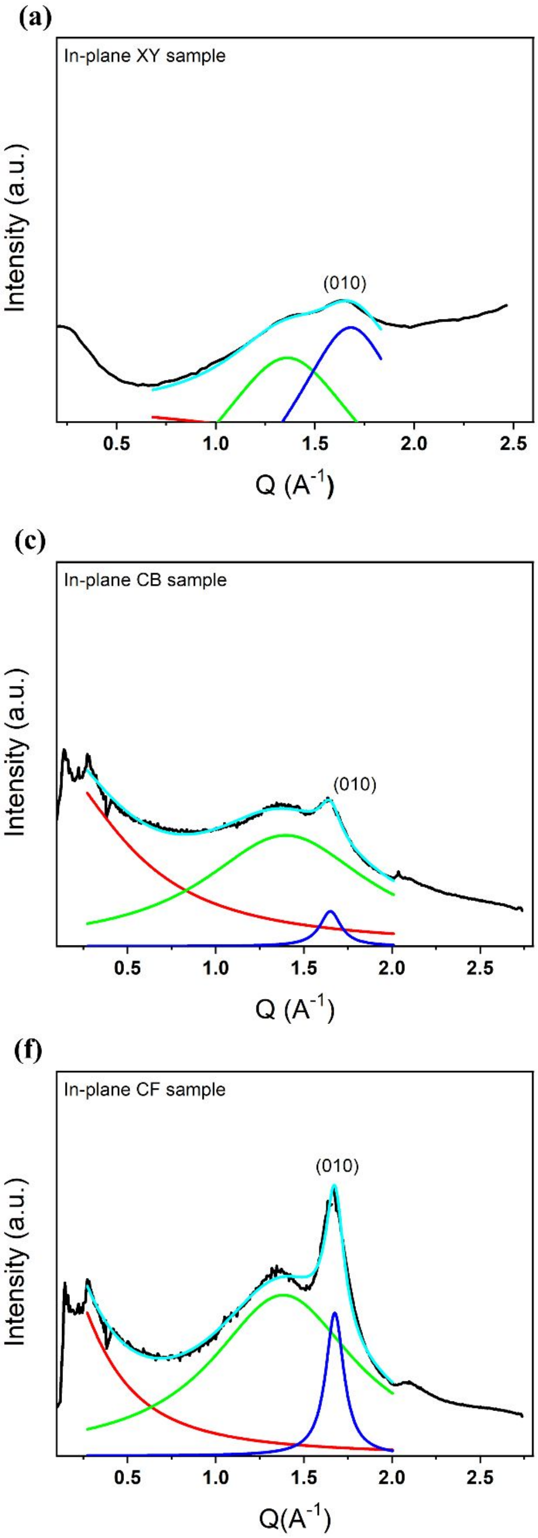

Figure S8. 1D in-plane linecut of (a) XY, (b) TOL, (c) CB, (d) DCB, and (e) CF.

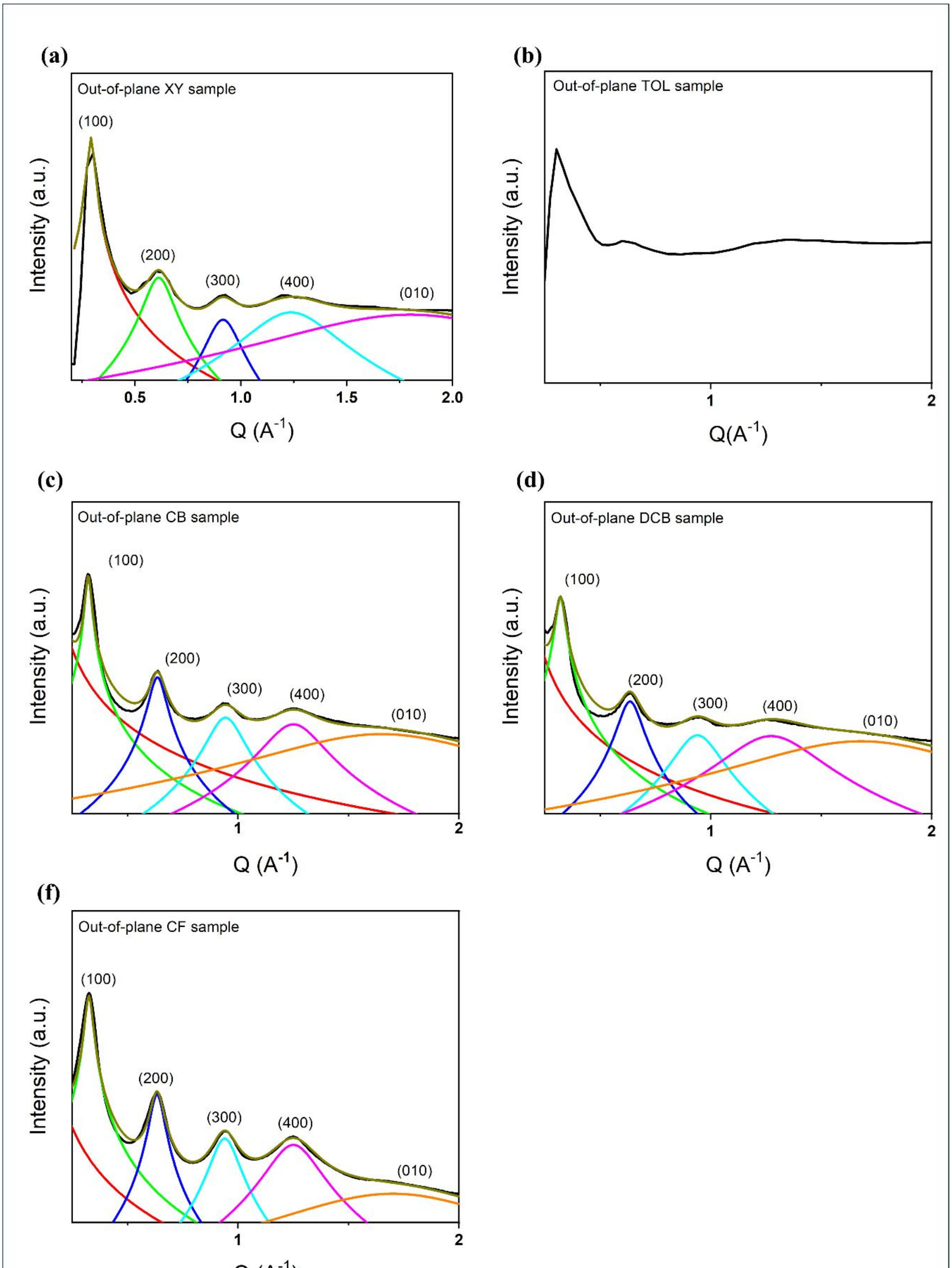

Figure S9. 1D out-of-plane linecut of (a) XY, (b) TOL, (c) CB, (d) DCB, and (e) CF.

Table S14. GIWAXS 1D linecut peak parameters

Sample		In-plane				Out-of-plane			
		q value (nm^{-1})	d-spacing (nm)	FWHM (nm^{-1})	g	q value (nm^{-1})	d-spacing (nm)	FWHM (nm^{-1})	g
(100)	XY	-	-	-	-	2.911	2.159	0.292	0.126
	TOL	-	-	-	-	-	-	-	-
	CB	-	-	-	-	3.222	1.950	0.250	0.111
	DCB	-	-	-	-	3.200	1.963	0.400	0.141
	CF	-	-	-	-	3.250	1.933	0.330	0.127
(010)	XY	16.800	0.374	0.400	0.195	18.000	0.349	12.000	0.326
	TOL	-	-	-	-				
	CB	16.504	0.381	1.434	0.118	16.500	0.380	10.000	0.311
	DCB	16.480	0.381	1.301	0.112	16.800	0.374	10.000	0.308
	CF	16.754	0.375	1.250	0.108	17.500	0.359	10.000	0.302

FWHM: Full width at half maximum

g: Paracrystallinity

Paracrystallinity was calculated from the equation below.[1]

$$g = \sqrt{\frac{\Delta q}{2\pi q_0}}$$

Where g is paracrystallinity, Δq is peak breadth, and q_0 is peak center position.

The details of the dataset, classification model, and regression model codes are available at the GitHub repository linked below.

https://github.com/hahaha144/Classification_modeling_DPPDTT_OFETs

Reference:

(1) Noriega, R.; Rivnay, J.; Vandewal, K.; Koch, F. P.; Stingelin, N.; Smith, P.; Toney, M. F.; Salleo, A. A General Relationship between Disorder, Aggregation and Charge Transport in Conjugated polymers. *Nat. Mater.* **2013**, *12* (11), 1038-1044. DOI: 10.1038/nmat3722.
